# 2D Material Nanofiltration Membranes: From Fundamental Understandings to Rational Design

**DOI:** 10.1002/advs.202102493

**Published:** 2021-10-19

**Authors:** Xiaopeng Liu, Ling Zhang, Xinwei Cui, Qian Zhang, Wenjihao Hu, Jiang Du, Hongbo Zeng, Qun Xu

**Affiliations:** ^1^ College of Materials Science and Engineering Zhengzhou University Zhengzhou 450001 P. R. China; ^2^ School of Chemical Engineering Zhengzhou University Zhengzhou 450001 P. R. China; ^3^ Institutes of Advanced Technology Zhengzhou University Zhengzhou 450052 P. R. China; ^4^ School of Metallurgy & Environment Central South University Changsha Hunan 410083 China; ^5^ Department of Chemical and Materials Engineering University of Alberta Edmonton Alberta T6G 1H9 Canada

**Keywords:** 2D material membranes, ion rejection, mechanistic understanding, nanofiltration, water flux

## Abstract

Since the discovery of 2D materials, 2D material nanofiltration (NF) membranes have attracted great attention and are being developed with a tremendously fast pace, due to their energy efficiency and cost effectiveness for water purification. The most attractive aspect for 2D material NF membranes is that, anomalous water and ion permeation phenomena have been constantly observed because of the presence of the severely confined nanocapillaries (<2 nm) in the membrane, leading to its great potential in achieving superior overall performance, e.g., high water flux, high rejection rates of ions, and high resistance to swelling. Hence, fundamental understandings of such water and ion transport behaviors are of great significance for the continuous development of 2D material NF membranes. In this work, the microscopic understandings developed up to date on 2D material NF membranes regarding the abnormal transport phenomena are reviewed, including ultrafast water and ion permeation rates with the magnitude several orders higher than that predicted by conventional diffusion behavior, ion dehydration, ionic Coulomb blockade, ion–ion correlations, etc. The state‐of‐the‐art structural designs for 2D material NF membranes are also reviewed. Discussion and future perspectives are provided highlighting the rational design of 2D material membrane structures in the future.

## Graphical abstract

Fundamental understandings regarding the abnormal water and ion transport phenomena through severely confined nanocapillaries (<2 nm) in 2D material nanofiltration (NF) membranes are reviewed. The state‐of‐the‐art structural designs for 2D material NF membranes are also highlighted and discussed based on the microscopic understandings, which inspires rational designs with the superior overall performance for water purification in the future.

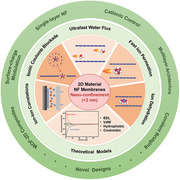

## 1 Introduction

With fast economic development and population growth, fresh water shortage and energy resource depletion have been two formidable challenges for decades. As such, water purification using energy‐efficient and cost‐effective manners is highly demanded.^[^ ^1^ ^]^ Conventional water purification methods like distillation are effective in removing minerals, bacteria, and water‐hardening substances; however, they are not efficacious in the removal of chlorine or volatile organic compounds, and suffer from high energy consumption.^[^ ^2^ ^]^ Hence, energy‐efficient, membrane‐based separation technologies have attracted great attention in the academic field and in industries for desalination, drinking water production, wastewater treatment, etc.^[^ ^3^ ^]^

Among various forms of membrane‐based separation technologies, reverse osmosis (RO) has been widely accepted as an effective and environmental‐friendly method for desalination.^[^ ^4^ ^]^ RO often operates under the influence of the osmotic pressure differences between saltwater and pure water with a high pressure applied onto the saltwater side.^[^ ^4b^ ^]^ Under standard conditions, the applied pressure can easily reach as high as over 60 bar for the normally used semipermeable membranes of nonporous polymeric membranes in RO process.^[^ ^5^ ^]^ Although the recent advances in polymer science and technology have reduced the energy requirement for the RO of water desalination,^[^ ^2a^ ^]^ these nonporous polymer‐based RO membranes still requires relatively high energy cost meanwhile showing inferior resistance to high temperature and pressure.^[^ ^6^ ^]^ Forward osmosis (FO) separation process, on the other hand, is simply driven by the osmotic gradient across a semi‐permeable membrane with low operational energy input; however, it is always coupled with other techniques, such as distillation and RO, for desalination,^[^ ^7^ ^]^ so that the high energy input is still inevitable. Besides the energy cost, these non‐porous polymer membranes also have drawbacks of relatively low water flux and deficient antifouling properties,^[^ ^8^ ^]^ significantly deteriorating the efficiency of water purification. Therefore, the sustainable development of membrane technologies is urgently needed.

**Figure 1:**
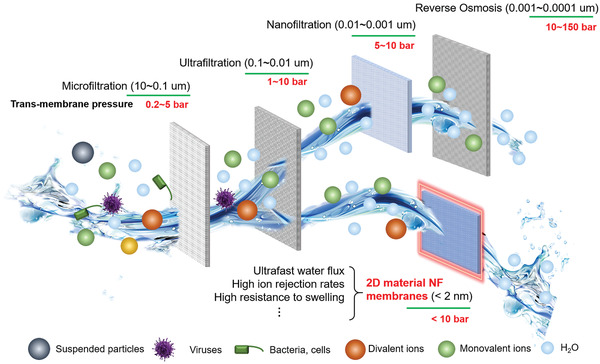
Schematic diagram showing the potential of 2D material NF membranes for water purification.

In general, an ideal RO or FO membrane should have high water flux, high rejection rates of ions, high resistance to swelling, mechanical stresses generated under operating conditions, as well as chemical and bio‐fouling,^[^ ^2^, ^8^, ^9^ ^]^ achieved in a constantly improved, energy‐saving method. Other than semipermeable and nonporous membranes mentioned above, nanofiltration (NF) membranes have thin and porous structures with the pore size normally in the sub‐2‐nanometer range, and thus, have advantages of low energy cost and high efficiency, e.g., low operating pressure and high water flux.^[^ ^10^ ^]^ However, the ion rejection rate has been traded off, at least to some degree, due to the presence of nanopores. Polymeric NF membranes have been reported to allow water molecules and/or monovalent ions to pass through while blocking particles and multivalent ions, leading to their ion rejection capability between nonporous polymeric membranes and ultrafiltration (normally > 10 nm pores) (**Figure** **1**).^[^ ^11^ ^]^ Since the ion rejection is mainly realized by size‐exclusion mechanism in NF membranes, reducing the pore size to exclude small ions may cause the reduction of water flux. In addition, pore geometry and pore chemistry are also important in determining the interaction environment during water and ion transport. Therefore, pore structures, including pore size, pore geometry, and pore chemistry, are the dominant factors that affect water and ion permeation processes,^[^ ^12^ ^]^ which should be carefully manipulated to balance the trade‐off between water flux and ion rejection rates, especially the rejection of monovalent salt ions for NF membranes.

Recently, considerable research efforts have been taken on the development of 2D material NF membranes for water purification, due to their tunable pore size, geometry and chemistry, good chemical resistance, sufficient mechanical strength, and superior antifouling properties.^[^ ^13^ ^]^ The attempted 2D materials include graphene,^[^ ^14^ ^]^ graphene oxides (GOs),^[^ ^15^ ^]^ reduced graphene oxides (rGOs),^[^ ^16^ ^]^ MXenes,^[^ ^17^ ^]^ layered transition metal dichalcogenide (TMDCs),^[^ ^18^ ^]^ 2D metal‐organic frameworks (MOFs),^[^ ^19^ ^]^ 2D covalent organic frameworks (COFs),^[^ ^20^ ^]^ etc. More importantly, the quickly expanding 2D material categories ^[^ ^21^ ^]^ provide us with a diversified materials platform for the maneuver of sub‐2‐nanometer pores to optimize the balance mentioned above for NF membranes. There are basically two forms of 2D material NF membranes, a single‐layer 2D material NF membrane and a restacked thin‐film NF membrane, if without considering the blending of 2D materials in polymeric membranes.^[^ ^22^ ^]^ In the former, the pore size can be controlled by the parameters of pore‐forming techniques, e.g., plasma etching time, hydrogen peroxide, and ozone treatment,^[^ ^2^, ^23^ ^]^ from which the pore geometry and pore chemistry have also been determined. In the latter, the restacked interlayer spacing forms the 2D slit pores (pore geometry) with the pore size that can be adjusted through inserted ions or small molecules.^[^ ^24^ ^]^ In this case, the pore chemistry is normally determined by the chemistry of 2D materials themselves and the surface modification methods used for the 2D materials.

Interestingly, different from polymeric nonporous and polymeric NF membranes, when constructing NF membranes using 2D materials with severely confined space of <2 nm, abnormal phenomena of permeation have been observed. For example, ultrafast water and ion permeation rates were detected to be several magnitudes higher than that predicted by conventional diffusion behavior in GO membranes, which is mainly attributed to a large capillary force (> 1000 Pa) generated within graphene nanocapillaries.^[^ ^25^ ^]^ This is particularly interesting because this finding suggests that reducing the pore size in membranes to exclude ions would not necessarily cause the reduction of water flux, and thus, it is highly possible to achieve fast water flux and high ion rejection rates at the same time using these nanocapillaries (Figure 1). Furthermore, some other unusual phenomena have also been reported in graphene nanocapillaries, such as ultra‐dense packing of ions, drastic changes in diffusion coefficients, etc.,^[^ ^26^ ^]^ showing the significance of studying ion and molecular transport behaviors within the highly confined space. In a broad view, these investigations would also render us with the guidance in the theoretical understanding and technical development in other related areas, including capacitive deionization, salinity gradient energy harvesting, electrochemical capacitors, and field‐effect transistors with gate dielectrics of ionic liquids.^[^ ^27^ ^]^

Regarding the above‐mentioned significance of 2D material NF membranes, in this review, we will focus on the important microscopic understandings of water and ion transport behaviors through 2D nanocapillaries (<2 nm) developed up to date; in addition, the effect of electric fields on the transport of water and ions in nanocapillaries will also be reviewed. Note that most of the work reviewed in this paper have both experimental evidence and molecular simulations. All the microscopic understandings point to the importance of tuning pore structures with angstrom precision. Therefore, we also highlight the newly developed designs, for the precise control of the interlayer spacing of restacked 2D material NF membranes, for the fine structural control of single‐layer 2D material NF membranes and MOF‐2D material composite NF membranes, and for the surface‐charge modulation of GO membranes. Further note that 2D material NF membranes have been evaluated not only under pressure‐driven NF mode but also under FO mode in the literature, both of which will be included in this review. For the sake of the completeness, we also covered the most recent advances in the rejection of organic dyes. Discussions will be made and future perspectives will be provided in terms of the rational design of 2D material membrane structures, based on the mechanistic understanding of superior water flux and ion rejection in the NF membranes.

## 2 Fundamental Understandings of Water and Ion Transport through sub‐2‐nm Capillaries

### 2.1 Characteristics of GO and GO Membranes

Water and ion transport behaviors in 2D nanocapillaries have been investigated broadly based on the restacked 2D material NF membranes,^[^ ^14^, ^28^ ^]^ among which GO membranes with 2D graphene nanocapillaries are the most extensively studied subject. GO is an oxidized form of graphene with a high density of oxygenated functional groups on its surface, e.g., carboxyl, epoxy, hydroxyl, and carbonyl groups. Note that the carboxyl and carbonyl groups are mainly reside at the edges of GO, while hydroxyl and epoxy groups are randomly distributed on the basal plane.^[^ ^29^ ^]^ Such abundant oxygenated functional groups on GO make the material very hydrophilic, in contrast to its un‐oxidized state, graphene.^[^ ^29a^ ^]^ Depending on the fabrication methods and parameters, the oxygen content on GO nanosheets can reach as high as over 40 wt%,^[^ ^30^ ^]^ and thus, it is well accepted that GO nanosheets have two distinguished regions in terms of the affinity to water molecules, hydrophobic pristine graphene regions as well as hydrophilic oxidized regions.^[^ ^31^ ^]^

**Figure 2:**
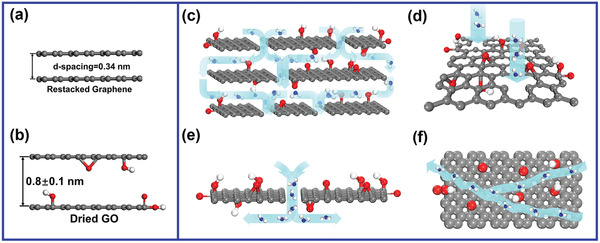
The illustrations of graphene nanocapillaries and transport pathways. a,b) *d*‐spacing of a a) pure graphene channel and b) a dried GO membrane. c–f) Three pathways to conduct water and ion permeation in the c) restacked GO membranes, including d) pore defects on the basal plane, e) interedge pores, and f) 2D slit pores in the interlayer spacing.

In GO membranes, GO nanosheets are restacked to form slit pores. The pore dimensions can be determined from the lateral size of GO and their restacked interlayer spacing (commonly termed as *d*‐spacing). *d*‐spacing is defined as the center‐to‐center distance between two adjacent graphene planes, which is normally measured by X‐ray diffraction (XRD). The *d*‐spacing of a pure graphene channel is 3.4 Å (**Figure** **2**a), and that of a dry GO membrane is ≈8 Å (Figure 2b). In calculations, the *d*‐spacing of GO membrane can be estimated according to the structure model shown in Figure 2b. The carbon‐oxygen hydrogen bond length is ≈2.2 Å and van der Waals radius of oxygen atoms is ≈1.7 Å.^[^ ^32^ ^]^ Twice the sum of these two values gives the total *d*‐spacing of 7.8 Å, which is very close to the measured value, suggesting that GO nanosheets are well restacked in the membrane maybe through the hydrogen bonding across the functional groups.^[^ ^32^ ^]^ In addition, since the interlayer spacing in the graphite configuration in Figure 2a is impermeable to any molecules ascribing to the delocalization of the electron clouds of the p orbitals and *π*‐*π* stacking,^[^ ^33^ ^]^ the interlayer spacing of ≈8 Å in the dry configuration of GO membranes would create a free spacing of ≈4.6 Å between two adjacent graphene planes, forming 2D hydrophobic graphene nanocapillaries in the pristine graphene region of GO membranes. The above chemical and structural information of GO membranes is very useful when analyzing the permeation performance of water and ions, or when constructing a suitable structural model for simulations.

Regarding transport through GO membranes (Figure 2c), water molecules or ions have three types of pathways, including those across the pore defect on the basal plane of GO nanosheets (Figure 2d), through the interedge area between two separated GO nanosheets aligned side by side (Figure 2e), and across the free space of interlayer spacing between two restacked GO nanosheets (Figure 2f).^[^ ^34^ ^]^ The defect pores are small in size (<1 nm), but they are difficult to be controlled uniformly for all the GO nanosheets in membranes. Interedge pores are normally larger than 1 nm depending on the lateral size of GO nanosheets and the fabrication process of the membranes, but still, their pore size is hard to manipulate. The interlayer spacing of GO membranes, however, can be controlled precisely in a restively large range from 0.34 to over 1 nm, although the measures to tune the interlayer spacing have been keeping improving constantly. Fortunately, water or ion transport through the free space of interlayer spacing is always the rate‐determining step, especially when GO membranes are thick enough. Therefore, GO membranes represent a unique experimental platform for the study of water and ion transport behavior through 2D graphene nanocapillaries (<2 nm).

In this perspective, the interlayer spacing of GO membranes plays a key role in determining their separation properties. From the discussion above, it seems that a GO membrane should possess an ideal structure for water/ion separation, since a free spacing of 4.6 Å in the interlayer spacing of GO membranes should be effective for blocking most hydrated salt ions (e.g., Na^+^ with a hydrated diameter of 7.2 Å)^[^ ^35^ ^]^ while allowing water molecules (≈2.5 Å) to pass. However, the presence of oxygenated functional groups on GO makes the material a high tendency to absorb water, causing its swelling in humid or aqueous environments. The swelling of GO membranes results in the expansion of *d*‐spacing, and in some cases, this value can reach as large as 6–7 nm, measured by quartz crystal microbalance with dissipation (QCM‐D).^[^ ^36^ ^]^ This expansion significantly changes the original structure of GO membranes in their dry configurations, and thus, deteriorates their target performance. Furthermore, the oxygenated functional groups on GO were reported to severely impede water flux,^[^ ^37^ ^]^ maybe due to the hydrogen bonding instantly forms or breaks during water transport. Unfortunately, if removing those oxygenated functional groups on GO to increase the pristine graphene region, for example, forming reduced GO (rGO), the 2D nanosheets would restack spontaneously to the original state of graphite with the *d*‐spacing being 3.4–3.6 Å and no free space.^[^ ^38^ ^]^ This will completely seal the 2D graphene nanocapillaries and prevent any substance from transmission through this type of pathway. Therefore, what is the optimum pore structure so that water flux and ion rejection rates can be improved simultaneously, and how to construct the optimized interlayer structures which are also sufficiently strong to prohibit swelling in water, are two fundamental questions with significant technical barriers to achieve. In this sense, it is of great importance in understanding water and ion transport through nanocapillaries microscopically, before analyzing the recent advances in the development of 2D material NF membranes and looking forward to the desired structural design of the membranes and the promising directions to overcome the challenges.

### 2.2 Water Transport in 2D Graphene Nanocapillaries (<2 nm)

The study of water transport within graphitic nanocapillaries can be traced back to the simulation and experimental investigations on the transmission of water molecules through carbon nanotubes (CNTs).^[^ ^39^ ^]^ Hummer et al.^[^ ^39a^ ^]^ used molecular dynamics (MD) simulations to show that, although CNT walls had strongly hydrophobic character, the initially empty central channel of the nanotube can be rapidly occupied by water molecules that formed a 1D ordered chain, due to the water‐nanotube van der Waals attractions. It was further demonstrated that water molecules not only penetrated into, but were also conducted through, the nanotube, with the water flow rate comparable to that flew through twice as long channel of the transmembrane protein aquaporin‐1.^[^ ^40^ ^]^ The water conduction was also found to follow pulse‐like transmission, and during those bursts, the water chain moved with little resistance through the nanotube, unhindered by their interactions with the hydrophobic wall.^[^ ^39a^ ^]^ Later on, Majumder et al.^[^ ^41^ ^]^ designed an array of aligned CNTs and presented a clear experimental evidence that water flew through those membranes was four to five orders of magnitude faster than that would be predicted from conventional fluid‐flow theory, approaching the rate that flew through biological channels. Similar phenomenon had also been observed by Holt et al.^[^ ^42^ ^]^ All the pioneering work established the abnormal fast water transport in the confined environment (<2 nm) and attributed this unusual phenomenon to the ordered hydrogen bonds between water molecules and the weak attraction between water and smooth carbon nanotube graphitic wall, resulting in almost frictionless and very rapid water flow inside CNTs.^[^ ^42^ ^]^ However, there was always experiment‐theory dispute concerning water permeation through CNTs^[^ ^43^ ^]^ and mechanisms were not fully disclosed based on CNT channels.

**Figure 3:**
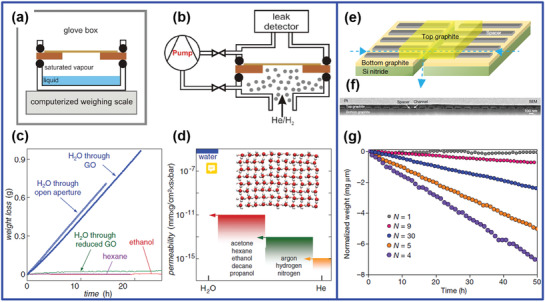
Water transport within 2D graphene‐based nanocapillaries. a) Schematic of the experimental setup for pervaporation test of GO membranes; b) permeation of helium and hydrogen studied by mass spectrometry. c,d) Permeation through GO: c) weight loss for a container sealed with a GO film; d) permeability of GO membranes with respect to water and various small molecules; schematic representation of the structure of monolayer water inside a graphene capillary with *d* = 7 Å using MD simulations (inset in (d)). a‐d) Reproduced with permission.^[^ ^38c^ ^]^ Copyright 2012, AAAS. e–g) Pure graphene nanocapillaries for pervaporation test: e) the illustration of graphene capillary devices; f) a scanning electron microscopic (SEM) image of a cross‐section of a device showing an array of nanocapillaries with cavity height about 15 nm; g) water flow through graphene channels of different heights, indicated by the number of graphene layers of *N*. e‐g) Reproduced with permission.^[^ ^25^ ^]^ Copyright 2016, Springer Nature.

When graphene was discovered by A. K. Geim and K. S. Novoselov, the topic of studying water transport within 2D graphene nanocapillaries almost attracted research attention immediately. Since GO membranes are easy to fabricate and possess pristine graphene regions (Figure 2f), GO membranes were first used to study this topic.^[^ ^38c^ ^]^ Nair et al.^[^ ^38c^ ^]^ tested water permeation behavior in GO membranes using a pervaporation setup (**Figure** **3**a). It was found that submicrometer‐thick GO membranes can be completely impermeable to most liquids, vapors, and gases, including helium, but allowed unimpeded permeation of water (Figure 3b). The measured permeation rates showed that water permeated through GO membranes at the rate at least 10^10^ times faster than He (Figure 3c). As discussed above, the free space between two graphene sheets in GO membrane is ≈4.6 Å, which is sufficient to accommodate a monolayer of water,^[^ ^44^ ^]^ and thus, it was proposed in the study that these empty spaces formed a network of hydrophobic graphene nanocapillaries that allowed the frictionless flow of the monolayer water leading to fast water flow. Further analyses were conducted using MD simulations.^[^ ^38c^ ^]^ It was demonstrated that, when *d* (*d*‐spacing) was in the range between 6 and10 Å, water rushed into the graphene nanocapillaries^[^ ^45^ ^]^ and formed a highly ordered monolayer shown in the inset image of Figure 3d. These findings were consistent with those observations found in CNTs.^[^ ^39^, ^42^ ^]^ More importantly, the simulations also indicated that the estimated capillary pressure involved in the intermediate *d* (6–10 Å) was on the order of 1000 bar, which was in qualitative agreement with the estimation by using van der Waals interaction energy between water and graphite.^[^ ^46^ ^]^ Therefore, although water‐graphene attraction is relatively weak, much weaker than that for water‐oxygenated functional groups on GO, the capillary pressure for water in the graphene nanocapillaries (<2 nm) is remarkably high, which makes the fast frictionless flow and the ordered structure of water molecules reasonable to understand.

Based on these studies, water transport behavior in graphene nanochannels should be dependent on their slit pore size. To clarify this point, fabrication of pure graphene nanocapillaries is highly desired in order to avoid the influence of oxygenated functional groups. The pure graphene capillary devices were successfully fabricated by Radha et al.,^[^ ^25^ ^]^ as shown in Figure 3e. Graphene nanosheets with controlled number (*N*) of layers were tightly compacted by upper and bottom graphite plates; hence, the slit pore size can be determined by the number graphene layers (*N*) stacked between (Figure 3e). The constructed graphene nanocapillaries was clearly demonstrated by electron microscopes, as shown in Figure 3f. It was showed (Figure 3g) that, as the free space in graphene nanocapillaries decreased from ≈10 nm (*N* = 30), the amount of evaporated water decreased (comparing *N* = 30 and *N* = 9), as generally expected. However, as the free space kept decreasing down to <2 nm (*N* = 4, 5), the amount of water evaporation shot up by more than an order of magnitude. MD simulations demonstrated that the capillary pressure can reach about 1000 bar at *N =* 2 or 3, and theoretical analyses attributed the dominant factor of this abnormal flux enhancement to be the disjoining pressure at small *N*, which was also related to van der Waals interactions between water molecules and graphene walls.^[^ ^47^ ^]^ Hence, the dependence of water permeation behavior on the slit pore size of graphene nanocapillaries obtained experimentally in this work^[^ ^25^ ^]^ correlated well with the simulation results done by Nair et al.^[^ ^38c^ ^]^ Although the device fabricated in this work was not practical to be used for water purification, it presented a clear experimental evidence for the unexpected enhancement of water flow in 2D graphene nanocapillaries with the slit pore size smaller than 2 nm.

With oxygenated functional groups on GO, water flow should be severely impeded, which has been confirmed by many reports.^[^ ^37^ ^]^ Simulations also implied that the water permeation would be strongly impeded for all *d* ≤ 10 Å by adding arrays of epoxy groups to graphene planes to mimic the oxidized graphene regions.^[^ ^38c^ ^]^ However, the phenomenon observed by Nair et al. using GO membranes^[^ ^38c^ ^]^ was in good accordance with that observed by Radha et al. using hydrophobic graphene nanocapillaries.^[^ ^25^ ^]^ It is believed that this agreement was achieved because of the use of pervaporation setup in the work of Nair et al. (Figure 3a,b). In the case of pervaporation, the swelling of GO membranes was prohibited and water molecules passing through the membranes were in the state of water vapor rather than in its liquid phase, so that water molecules could choose the most favorable pathway through the interlayer spacing. Touching this point, it made researchers still doubt about the preservation of fast water flux through 2D graphene nanocapillaries (<2 nm) in pressure‐driven permeation. Since GO membranes were found to show a very low steady‐state water permeation rate of 0.05 L m^−2^ h^−1^ bar^−1^ in a dead‐end filtration test, it was suspected that the capillary pressure did not exist under the pressure‐driven permeation mode.^[^ ^48^ ^]^ The clarification of this point by experiments is of great significance, which may require a special design of graphene‐based NF membranes in the future.

**Figure 4:**
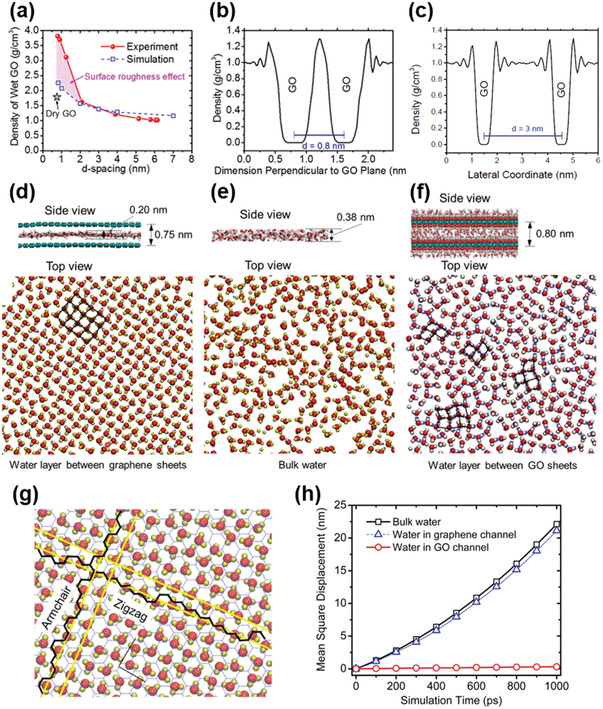
MD simulation results for GO membranes soaked in pure water. a) Densities of a wet GO membrane with different *d*‐spacings. b,c) Density profile of water between GO sheets with a *d*‐spacing of b) 8.0 Å and c) 3 nm. d) Side and top views of 1250 water molecules that formed a well‐aligned rhombus‐shaped crystal layer in a graphene channel with a *d*‐spacing of 7.5 Å, e) a typical hexagonal structure of ice crystals in bulk water, and a f) less aligned rhombus‐shaped crystal layer in a GO channel with a *d*‐spacing of 8.0 Å. g) Alignment of water molecules along the armchair and zigzag directions of the graphene carbon lattice. h) Mean square displacements of water molecules in bulk, the graphene channel, and a GO channel, respectively. Reproduced with permission.^[^ ^36^ ^]^ Copyright 2017, American Chemical Society.

From the discussion shown above, it is also noted that the fast frictionless flow and the ordered structure of water molecules within graphene nanocapillaries (<2 nm) are highly correlated. This is reasonable because a large number of water molecules can be attracted into the graphene nanocapillaries by the large capillary pressure, forming a water layer with the density higher than that of the bulk water, and thus, an ordered structure is resulted. This understanding is also consistent with previous studies, in which the highly confined environment at the nanoscale can disrupt the hydrogen‐bonding network in water and create new polymorphs of quasi‐2D (Q2D) ice.^[^ ^47b^ ^]^ With assistance of QCM‐D, Zheng et al.^[^ ^36^ ^]^ might present experimental proofs regarding this issue. It was found that, when soaking GO membranes in water completely, the membrane thickness did not increase abruptly at the beginning, indicating that it took longer for the intercalated water molecules to push the adjacent GO nanosheets away from each other and to cause swelling. Hence, the investigation of the density of the water layer at the beginning stage of soaking could give us the structural information of the water layer within the graphene nanocapillaries. The measurements indeed showed that a wet GO membrane at the beginning of soaking had a density as high as over 2.3 g cm^−3^ (**Figure** **4**a). It meant that by absorbing water into the severely confined space before the expansion of the *d*‐spacing, a wet GO membrane was actually denser than both a dry GO membrane (1.79 g cm^−3^) and bulk water (1.0 g cm^−3^).^[^ ^36^ ^]^ Therefore, it strongly supported that water molecules were attracted into the graphene nanocapillaries (<2 nm) by the large capillary pressure, with the number of inserted water molecules higher than that in the same volume of bulk water, resulting in the structure of water molecules within the nanocapillaries more ordered than that in the bulk water.

MD simulations showed that the density of the intercalated water decreased with increasing *d*‐spacing (Figure 4b,c), corresponding well with the experimental results in Figure 4a.^[^ ^36^ ^]^ In addition, the structures of water were simulated between hydrophobic graphene nanocapillaries (Figure 4d), within bulk water (Figure 4e), and between oxidized graphene nanocapillaries (Figure 4f). Only the pristine graphene‐sandwiched water demonstrated a crystal feature with the highest density among all three structures, consistent with the simulation results reported previously.^[^ ^25^, ^38^ ^]^ Furthermore, this crystal structure had a high correlation with the graphene lattice, as shown in Figure 4g, while the alignment of the water structure was obviously disturbed by the extruding oxygenated functional groups from the oxidized graphene planes (Figure 4f).

Interestingly, despite the density difference, the mobility of water molecules in the hydrophobic graphene nanocapillary was found to be almost the same as that in the bulk water, both of which were much higher than that in the oxidized graphene channel (Figure 4h), which coincided with the concept of frictionless flow of water molecules within graphene nanocapillaries (<2 nm).^[^ ^36^ ^]^ Although interesting, the underlying reason of this high mobility was not presented in the study, especially considering that the intercalated water molecules were present under such a highly pressurized environment, leading to the unresolved dispute for the case of water transport under the pressure‐driven permeation mode. Boukvalov et al. used MD simulations to show that the migration energy barrier for bi‐layer ice (ordered water) sliding was an order of magnitude lower than that of monolayer ice sliding,^[^ ^49^ ^]^ suggesting a possible direction to explain the high mobility of the ordered water structure.

Therefore, although oxygenated functional groups on GO nanosheets obscured our observations in some cases, both experimental evidence and simulation results had already been demonstrated to confirm that, 1) water transport in 2D graphene nanocapillaries (<2 nm) is anomalously fast, and 2) the density of water in this severe nanoconfinement is higher than that of bulk water with an ordered structure.^[^ ^25^, ^36^, ^38^ ^]^ Simulations attributed the unusual phenomena to be the large capillary pressure (≈1000 bar) arising from the van der Waals attraction between water molecules and graphene walls.^[^ ^25^ ^]^ Therefore, the construction of the membranes possessing 2D hydrophobic graphene nanocapillaries with the *d*‐spacing finely tuned below 2 nm is highly desired to achieve fast water flux, high ion rejection rates, and good antiswelling properties. However, as discussed in Section 2.1, technical barriers exist due to the easy restacking of rGO nanosheets.

### 2.3 Ion Transport in 2D Graphene Nanocapillaries (<2 nm)

In achieving the optimum balance between water flux and salt ion rejection, the investigation of ion transport behavior in graphene nanocapillaries is also very crucial.^[^ ^50^ ^]^ Considering the high capillary pressure in graphene nanocapillaries (<2 nm), similar behavior is expected for ion transport as that for water transport; however, it may differ, at least to some extent, due to the following aspects, 1) salt ions are solutes in liquid water phase, and thus, the water structure within the confined regions may change because of the inclusion of ions; 2) the interaction between salt ions and graphene sheets is different from that between water molecules and graphene sheets; 3) the effect of water‐ion interaction, e.g., hydration energy, should also be included when conducting ions through the graphene nanocapillaries. In addition, the pervaporation setup would not be suitable for this study; instead, it requires complete soaking of membranes in aqueous solutions. Therefore, situations become more complicated for studying ion transport than that for water transport. Although the aspects listed above have not been fully disclosed, microscopic understandings of ion transport in graphene nanocapillaries (<2 nm) have already made significant progress.

**Figure 5:**
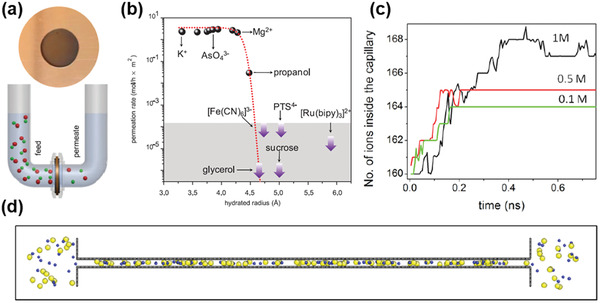
Ion permeation through GO laminates. a) A photograph of a GO membrane covering a 1‐cm opening in a copper foil and a schematic of the experimental setup. b) Sieving through the atomic‐scale mesh: no permeation could be detected for the solutes shown within the gray area during measurements lasting for at least 10 days. c,d) Simulated salt‐absorption effect: number of ions inside a 9 Å wide capillary (two layers of water) as a function of simulation time. Initial concentrations of NaCl in the two reservoirs are 0.1, 0.5, and 1 m for the different curves. c) The initial NaCl concentration inside the capillary is 1 m for all the curves; d) snapshot for the case of a 1M NaCl solution inside the capillary and 0.1 m in the reservoirs. Despite the large concentration gradient, ions move from the reservoirs into the capillary. Reproduced with permission.^[^ ^51^ ^]^ Copyright 2014, AAAS.

Joshi et al. first fabricated GO membranes with high stability in aqueous solutions.^[^ ^51^ ^]^ They claimed that, although those GO membranes could swell from 9 to 13 Å, they would be stable afterward, making the complete soaking test meaningful for the study of water and ion transport, as shown in **Figure** **5**a. The *d*‐spacing of 13 Å can be translated to 9–10 Å slit pore size (or free space of the graphene nanocapillary). As shown in Figure 5b, molecules or ions with the hydrated radius larger than 4.5 Å could be completed blocked by GO membranes, proving the accuracy of the pore size calculated. Furthermore, the water flow rate, in this case, was found to be around 0.2 L m^−2^ h^−1^ under the osmotic pressure of 25 bar at room temperature, which was significantly lower than that of the evaporation rate (≈10 L m^−2^ h^−1^).^[^ ^38c^ ^]^ Previous pervaporation test showed that water flow rates in graphene nanocapillaries (<2 nm) were as fast as their evaporation rates,^[^ ^38c^ ^]^ and thus, the reduction of water flow rates in GO membranes in the complete soaking test should be caused by the oxygenated functional groups that impeded the water flow. However, ion permeation rates (for hydrated ions with the radius smaller than 4.5 Å, e.g., Na^+^) through GO membranes were determined to be several thousands of times faster than those predicted from traditional ion diffusion model (Figure 5b), demonstrating similar transport behavior as that of water through graphene nanocapillaries (<2 nm) in the pervaporation test.^[^ ^25^, ^38^ ^]^ This unusual phenomenon can also be attributed to a capillary‐like pressure that acted on ions within a water medium.^[^ ^51^ ^]^ The model of MD simulations was established as shown in Figure 5c, and it was found that, when keeping the concentration of NaCl inside the graphene nanocapillary to be 1 m and changing the concentration of NaCl in two reservoirs from 0.1 to 1 m, there was always an influx of NaCl from the reservoirs into the capillary, despite the lower concentrations in the reservoirs (Figure 5d). If keeping the concentration in the reservoirs to be 0.1 m and increasing the concentration inside of the capillary, at 3 m, NaCl started to leave the capillary. This concentration gradient allowed the estimation of the capillary pressure to be on the order of 50 bar.^[^ ^51^ ^]^ Note that this value is much lower than that of the capillary pressure for water transport (1000 bar). The reason and the consequences of this capillary pressure reduction are still unclear. Moreover, although fast ion permeation rates were reported, whether these rates were underestimated because of the presence of oxygenated functional groups on GO is still debatable.

**Figure 6:**
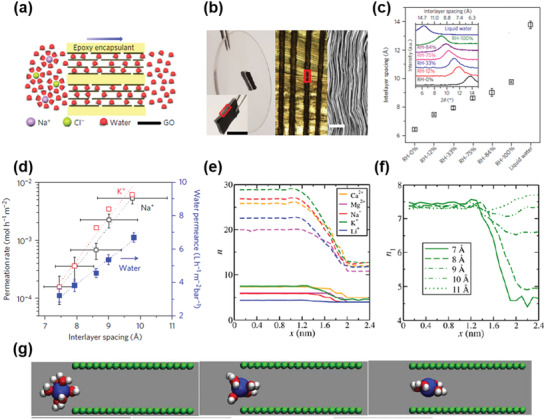
Ion dehydration. a–c) Physically confined GO membranes with tunable interlayer spacing: a) a schematic illustration of the direction of ion/water permeation along graphene planes; b) photographs of a membrane glued into a rectangular slot within a plastic disk of 5 cm in diameter, the cross‐sectional area marked by a red rectangle, and its SEM image; c) humidity‐dependent interlayer spacing, *d*, found using XRD (inset). d) Tunable ion sieving: Permeation rates for K^+^ and Na^+^ depend exponentially on the interlayer distance (left axis). Water permeation varied only linearly with *d* (blue squares, right axis). e,f) Simulations for the change of ion hydration number (*n*) during ion permeation through the 2D nanocapillary: e) the decrease in *n*_1_ (solid line) and *n*_2_ (dashed line) as the ions enter a channel with an interlayer spacing of 7 Å; f) *n*_1_ for K^+^ entering channels with interlayer spacing ranging from 7 to 11 Å. g) Dehydration of Mg^2+^: Mg^2+^ (blue) with the first hydration shell entering the 7 Å graphene channel (green) at *x* = 1.6, 1.8, and 2.0 nm in the simulation box (left to right). Reproduced with permission.^[^ ^52^ ^]^ Copyright 2017, Springer Nature.

From Figure 5c, it is also clear to see the salt ions underwent partial dehydration when entering the narrow graphene capillary. Abraham et al. investigated this phenomenon in depth (**Figure** **6**a),^[^ ^52^ ^]^ by controlling the interlayer spacing of GO membranes using relative humidity (Figure 6c), and meanwhile restricting its swelling by physically fixing the GO membranes in epoxy (Figure 6b). The cross section of those GO membranes was exposed for testing. The permeation rates were high for Li^+^, K^+^, and Na^+^ when *d*‐spacing was larger than 9 Å (Figure 6d), which was consistent with the observation in Figure 5b. However, these ion permeation rates decayed exponentially with the decreasing *d* (red and black lines in Figure 6d), while the linear reduction was found for water transport (blue line in Figure 6d). This was surprising because the *d*‐spacing of 9 Å can be translated to the free space of <6 Å. If only size exclusion mechanism had taken effect in this case, all hydrated ions of Li^+^ (7.64 Å), K^+^ (6.64 Å), and Na^+^ (7.16 Å)^[^ ^35^, ^53^ ^]^ should be excluded. Because of these experimental results, partial dehydration of ions was suggested and had then been investigated comprehensively.^[^ ^52^ ^]^ MD simulations showed that all five most widely used cations (Li^+^, Na^+^, K^+^, Ca^2+^, and Mg^2+^) were demonstrating partial dehydration process when entering narrow graphene nanocapillaries, where *n* is the number of water molecules in all hydration shells and *n*_1_ is that in the first hydration shell (Figure 6e). The degree of dehydration was also dependent on the *d*‐spacing of the capillaries (Figure 6f). Furthermore, for divalent cations, the partial dehydration could undergo a hydration shell reconfiguration process in order for the hydrated ions to fit the 2D geometry of the capillary (Figure 6g). Definitely, energy barriers are associated with these partial dehydration processes, which depend on valence, charge density of cations, *d*‐spacing of capillaries, etc. It was claimed that it was the partial dehydration energy barriers that caused the exponential decay of ion permeation rates with the decreasing *d*‐spacing, as indicated by the red and black lines in Figure 6d.^[^ ^52^ ^]^

Despite the progress of clarifying abnormal fast ion permeation rates and ion dehydration process, massive efforts are still needed to fully understand the mechanisms involved in the ion transport processes in 2D graphene nanocapillaries. However, the mechanistic studies are challenging because of the complexities introduced by the crowded colloidal forces under severe nanoconfinement, various types of ions, and the partial dehydration of ions. More importantly, water and ion transport behaviors in the nanocapillaries (<2 nm) made of other 2D materials are also interesting, which requires further studies in the future.

### 2.4 Effect of Electric Fields on Water and Ion Transport through Nanocapillaries

Electric fields arising from either externally applied potential or surface charge can strongly affect water and ion diffusion.^[^ ^54^ ^]^ The electrical bias can be applied across the membrane (external potential) or directly on the membrane (surface charge). Surface charge can also be generated where 2D nanosheets are functionalized with charged groups, e.g., negatively charged GO nanosheets or MXene nanosheets.

**Figure 7:**
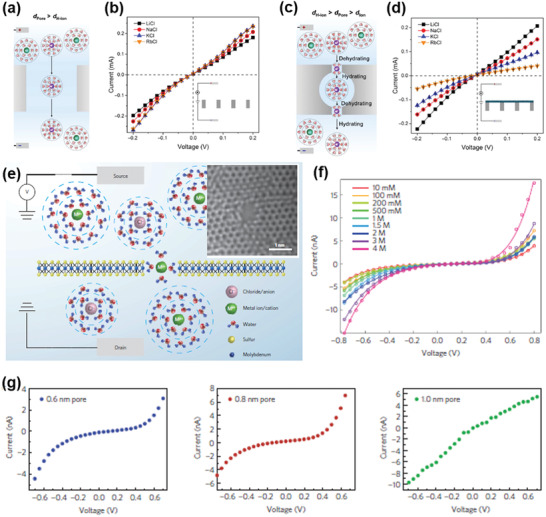
a) Schematic of ion transport through a pore with a diameter much larger than the hydrated ionic diameter, such as the 200‐nm porous AAO support. b) Current–voltage (*I*–*V*) characteristics of an AAO support measured with different ions. c) Schematic of ion transport through a simplified sub‐nm ZIF‐8 pore with 3.4‐Å‐diameter windows. d) *I*–*V* curves of ZIF‐8/GO/AAO membranes measured with different ions. a‐d) Reproduced with permission.^[^ ^55a^ ^]^ Copyright 2018, AAAS. e–g) Ion transport through the sub‐nm nanopore on a monolayer MoS_2_ nanosheet: e) schematic of single‐ion transport through a sub‐nm MoS_2_ nanopore; aberration‐corrected transmission electron microscopic (TEM) image of a 0.6‐nm‐diameter MoS_2_ nanopore (inset in e); f) *I*–*V* of a 0.6‐nm MoS_2_ nanopore in a KCl aqueous solution under different ion concentrations; g) variation of the linear–nonlinear transition with changing pore size, 0.6, 0.8, and 1 nm. The gap size decreases with the pore size, and disappears when the pore size reaches 1 nm. e‐g) Reproduced with permission.^[^ ^55b^ ^]^ Copyright 2016, Springer Nature.

Under externally applied potential, the partial dehydration of ions has also been reported on the NF membranes other than GO membranes.^[^ ^55^ ^]^ Zhang et al.^[^ ^55a^ ^]^ found that, when a very small voltage (20 mV) was applied across the anodic alumina oxide (AAO) membranes substrate with 200 nm straight pores (**Figure** **7**a), the order of ion transport was Rb^+^ > K^+^ > Na^+^ > Li^+^ with the highest ionic conductance being obtained for Rb^+^ (Figure 7b). Noted that the size of the hydrated cations follows the order of Li^+^(7.64 Å) > Na^+^(7.16 Å) > K^+^(6.64 Å) > Rb^+^ (6.58 Å).^[^ ^53^ ^]^ Smaller hydrated cations mean more cations passing through under the same electrical bias, and thus, the trend of the ionic conductance shown in Figure 7b is resulted and reasonable to understand. After the growth of ZIF‐8[Zn(2‐methylimidazolate)_2_]/GO layer on the AAO support with the pore size of ZIF‐8 being as small as 3.4 Å in diameter (Figure 7c), the alkali metal cations could also transport through the MOF membrane. The rates of ion transport in Figure 7d were comparable to those obtained in the case of Figure 7b, especially for Li^+^ cations.^[^ ^55a^ ^]^ This finding was a strong proof that the hydrated alkali metal cations underwent partial dehydration that facilitated their transmission through the MOF membrane. Interestingly, in contrast to the trend shown in Figure 7b, the order of ion transport in Figure 7d showed a reversed trend, Li^+^ > Na^+^ > K^+^ > Rb^+^ with the highest ionic conductance being obtained for Li^+^. Noted that the size of the dehydrated cations follows the order of Li^+^(1.2 Å) < Na^+^(1.90 Å) < K^+^(2.66 Å) < Rb^+^ (2.96 Å).^[^ ^53^ ^]^ Therefore, using MD simulations, it was revealed that the reversed order of ion transport through the MOF membrane (3.4 Å pore) was attributed to the reversed trend of cation size after dehydration.^[^ ^55a^ ^]^ The illustration of this situation with partial dehydration was also presented, as shown in Figure 7c. The partial stripping of the hydrated alkali metal cations was further confirmed by investigating the alkali metal ion selectivity on GO/AAO membrane under the same electric field. The interlayer spacing of GO layer in water was about 13.5 Å in their work, which was large enough to adopt all the hydrated alkali metal cations, and therefore, GO/AAO membrane did not demonstrate the same trend that shown in Figure 7d for ZIF‐8/GO/AAO membrane.^[^ ^55a^ ^]^

The partial dehydration of ions through 6 Å‐diameter nanopores has also been observed by Feng et al. on a monolayer MoS_2_ under externally applied potential.^[^ ^55b^ ^]^ The ion transport process was illustrated in Figure 7e with a nanopore shown in its inset image. All the regularly tested ions could pass through this pore when the electrical potential was applied above a critical value across the membrane. An example for the testing results in KCl solution was shown in Figure 7f, proving that the hydrated K^+^ (6.64 Å) underwent partial dehydration during their transmission across the nanocapillaries (6 Å). However, different from the ion transport behavior shown in the MOF membrane in Figure 7c,d, it was found in the study of porous monolayer MoS_2_ that the *I*–*V* characteristic curves exhibited a striking nonlinear behavior, with an apparent gap of ≈400 mV for KCl solution (Figure 7f). This unusual phenomenon was attributed to ionic Coulomb interaction between the dehydrated ions and pore walls.^[^ ^55b^ ^]^ The Coulomb gap could be described by a function of pore geometry and ion valence, using a capacitor model.^[^ ^56^ ^]^ In addition, using simulations, it was claimed that, similar to ion dehydration (Figure 7d), this ionic Coulomb blockade was also an energy barrier for ions to pass through the nanocapillaries. When the pore size was smaller than 6 Å, the ion dehydration energy was the main contributor to the energy barrier, while Coulomb energy was dominant in the pore size ranging from 6 to 10 Å. The voltage gap disappeared when the pore size was larger than 10 Å (Figure 7g).^[^ ^55b^ ^]^ It is very interesting that no obvious energy barrier was observed for the partial dehydration of ions in the study shown in Figure 7a–d. Although the underlying reason is still unknown, comparing previous studies we notice that, the energy barrier originated either from ion dehydration or from ionic Coulomb blockade is highly dependent not only on pore size but also on pore chemistry and pore geometry. In the study of the porous monolayer MoS_2_ (Figure 7e–g),^[^ ^55b^ ^]^ the functionalization of the pore surface by the pore‐forming process may play a key role in unveiling the phenomenon of ionic Coulomb blockade,^[^ ^57^ ^]^ while the case is different for the MOF membrane in Figure 7a–d.

**Figure 8:**
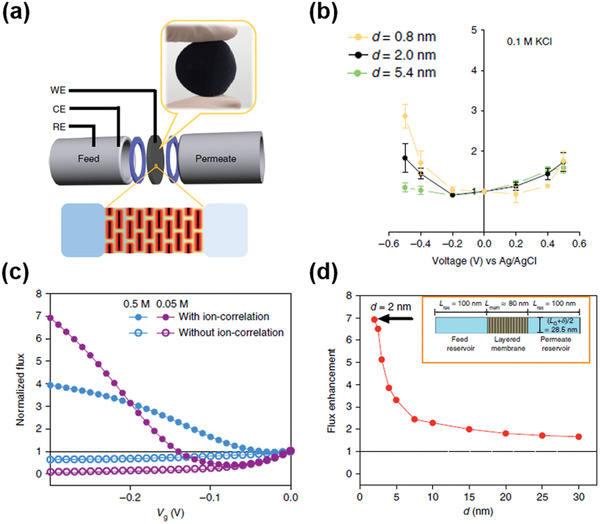
Ion–ion correlation. a) Schematic diagram showing the experimental setup for the investigation of ion diffusion through nanoconfined EDLs in charged, layered graphene‐based nanoporous membranes with various *d*. b) Normalized membrane flux dependence on *V*_g_ under various levels of nanoconfinement at 0.1 m KCl. c,d) Role of ion–ion correlations: the simulated membrane flux versus *V*_g_ relationships in 2 nm graphene nanoslits, obtained from c) continuum simulation based on the PNP model (circles) and the PNP/IC model (dots); d) the effect of *d* on flux enhancement simulated with *V*_g_ = −0.3 V and a feed concentration of 0.05 m. The inset in (d) shows the cascading graphene nanoslits model geometry used for simulation. Reproduced with permission.^[^ ^26^ ^]^ Copyright 2018, Springer Nature.

In the case of surface charge, when the slit pore size of 2D nanocapillaries are small enough so that the electrical double layers (EDLs) of the capillary walls overlap, direct electrostatic manipulation of ions across the nanocapillaries becomes possible.^[^ ^58^ ^]^ In particular, Cheng et al.^[^ ^26^ ^]^ investigated this ion manipulation under the nanoconfinement of <2 nm by applying electrical bias directly onto the chemically converted graphene membranes, as shown in **Figure** **8**a. The ion flux should be reduced when a negative potential was applied to the membrane (circles in Figure 8c), as predicted by the classical Poisson‐Nernst‐Planck (PNP) model. However, the experimental results showed the opposite trend, as shown in Figure 8b. It was found that, the ion flux increased almost three times at the negative potential of −0.5 V as respect to that at 0 V for the graphene nanocapillaries with the *d*‐spacing of 8 Å. This abnormal enhancement of ion flux could be explained by the short‐range, ion‐specific ion–ion correlations. The ion correlation (IC) was used to describe unconventional interfacial phenomenon, such as “overscreening”^[^ ^59^ ^]^ and “charge inversion”.^[^ ^60^ ^]^ Considering the extreme nanoconfinement (<2 nm) in this case, it was reasonable to incorporate this IC phenomenon into the classical PNP model, generating a PNP‐IC model. Using this new model, the abnormal phenomena observed in this study could be reproduced by the simulation results, as shown in the dotted lines in Figure 8c,d. Hence, this simulation suggested that such ion–ion correlations might play a key role in modulating the ion transport behavior between the surface‐charged capillary walls under severe nanoconfinement (<2 nm).^[^ ^26^ ^]^

The investigation of ion transport behavior through MXene‐based, 2D material NF membranes with the electrical bias directly applied on the membranes was also conducted.^[^ ^61^ ^]^ Ren et al.^[^ ^61a^ ^]^ found that the ion flux was enhanced by applying a positive potential to the membrane, while it was suppressed when a negative potential was applied. This phenomenon can be explained by the variation of *d*‐spacing for the restacked nanosheets upon electrical field. Since Ti_3_C_2_‐MXene nanosheets were negatively charged, when the positive potential applied, anions could be intercalated into the interlayer spacing, resulting in the expansion of the *d*‐spacing and the enhancement of ion flux. The case was reversed when the negative potential was applied.^[^ ^61a^ ^]^ Therefore, comparing water and ion transport behaviors in graphene nanocapillaries and those in MXene nanocapillaries under similar conditions, it is further confirmed that pore chemistry is critical besides slit pore size.

Although the disclosure of ion dehydration, ionic Coulomb blockade, and ion–ion correlations for the confined‐space transport under electric fields, the study of how the electric fields manipulate ion transport when coupled with severe nanoconfinement (<2 nm) is still an emerging area. Significant research interests are expected due to its broad implications in various areas including water treatment, batteries and supercapacitors, field‐effect transistor, etc.^[^ ^27^ ^]^

### 2.5 Theoretical Considerations of Water or Ion Transport through 2D Nanocapillaries

**Figure 9:**
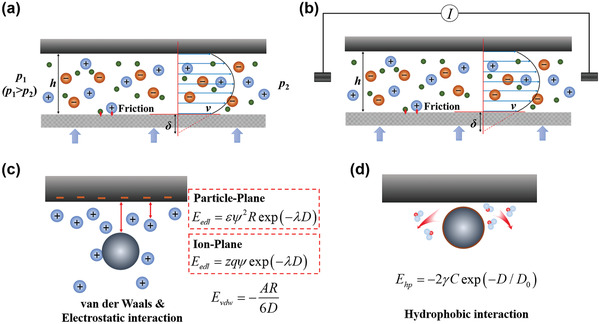
Illustration of three types of forces that should be considered when dealing water or ion transport in nanocapillaries. a) Pressure gradient with friction. b) Electrical potential gradient with friction. c,d) Various colloidal forces. *δ*, slip length; *h*, effective slit pore size; *v*, velocity distribution of water (or solution) flow. Approximate expressions in (c,d) for the free energy (*E*) of the colloidal interaction between a negatively charged spherical particle of radius *R* and a planar surface, and a small ion of charge +*zq* and a planar surface, as a function of the distance *D* between the particle (or ion) and the surface. *ψ*, electrical surface potential of surface and spherical particle; *λ*, Debye length; *ε*, medium dielectric constant; A, Hamaker constant; *γ*, interfacial tension between water (or solution) and the surface; C and D_0_ are constants. (See details from refs. [62] and [63].)

The transport phenomena in 2D capillaries were mainly treated with a theory based on continuum and mean‐field approaches; however, special attention should be given to the few layers of water or ions closest to the capillary wall. In general, there are three types of forces that should be considered when dealing water or ion transport in nanocapillaries.^[^ ^50b^ ^]^ First, the presence of external forces, such as a pressure gradient (**Figure** **9**a) and/or an electrical potential gradient (Figure 9b). Second, the presence of various colloidal forces, e.g., electrostatic forces between ions and surface‐charged walls (described as EDL forces), van der Waals force, and hydrophobic force (Figure 9c,d). Third, the presence of friction forces between the wall and water/ions (Figure 9a,b).^[^ ^50b^ ^]^ As discussed in the sections above, it is colloidal forces and friction forces that result in the unusual transport phenomena under severe nanoconfinement (<2 nm) observed experimentally. By analyzing all these forces, water or ion transport fluxes can be deducted theoretically.

#### 2.5.1 Antiswelling Properties

**Figure 10:**
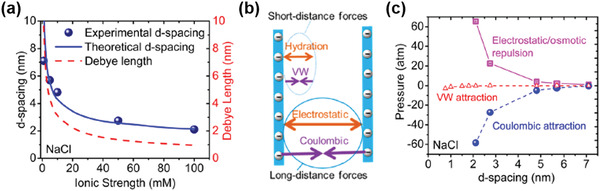
Theoretical analyses of antiswelling properties. a) Experimental and theoretical *d*‐spacing as well as Debye length of a GO membrane at equilibrium in NaCl solutions of different ionic strengths. b) Schematic illustration of repulsive and attractive forces between GO sheets. c) van der Waals and Coulombic attractions (negative value) and electrostatic/osmotic repulsion (positive value) between two GO sheets with different *d*‐spacings in a 1 × 10^−3^–100 × 10^−3^ m NaCl solution. Reproduced with permission.^[^ ^36^ ^]^ Copyright 2017, American Chemical Society.

Before going to water or ion fluxes, theoretical considerations of antiswelling properties of 2D material NF membranes should be reviewed first, which may be achieved by the analysis of colloidal forces within GO capillaries. Zhang et al. made a detailed investigation of colloidal forces in GO membranes after wetting by NaCl solution (**Figure** **10**).^[^ ^36^ ^]^ Because of the negatively charged surface, a GO nanosheet was associated with an electrostatic double layer (Figure 9c) in NaCl solution, from which the Debye length (*λ*) could be calculated, showing a general trend that *λ* decreases with increasing ionic strength, as illustrated (red curve) in Figure 10a. In particular, for a highly diluted solution of 1 × 10^−3^ m NaCl, the experimental *d*‐spacing of 7.1 nm was smaller than twice the corresponding *λ* of 9.6 nm, implying a significant overlap of EDL between two adjacent GO nanosheets.^[^ ^36^ ^]^ Without GO wetting in NaCl solution, the interactions between two negatively charged GO surfaces can be described by Derijaguin, Landau, Verwey, Overbeek (DLVO) theory, in which van der Waals attraction (Figure 9c) are balanced by electrostatic repulsion between two charged GO surfaces.^[^ ^64^ ^]^ When wetting, however, other non‐DLVO forces should be added for the analyses, including Coulombic attraction^[^ ^65^ ^]^ between negatively charged GO surface and positively charged cations, as well as short‐distance hydration force^[^ ^66^ ^]^ (Figures 9d and 10b). It was found that the dominant repulsive force of the electrostatic repulsion in this case was balanced by Coulombic attraction, rather than van der Waals attractive force (Figure 10c). The simulated *d*‐spacing fitted well with the experimental data, as shown in Figure 10a (blue line), suggesting the suitability of the model. This is a typical example where the considerations of pore size, pore chemistry, and pore geometry were all included in the theoretical model, which also provides us with theoretical guidance in improving antiswelling properties of GO membranes.

#### 2.5.2 Water Transport in 2D Capillaries

When interpreting water or ion fluxes through 2D nanocapillaries, driving forces should be analyzed. In terms of water transport in 2D capillaries, the driving forces can be an axial pressure gradient (hydrodynamic flow) and/or an axial electrical potential gradient (electro‐osmotic flow for solutions).^[^ ^47^, ^67^ ^]^ In a classical model where no external electrical potential is applied, water transport behavior can be described by Hagen‐Poiseuille flow equation with non‐slip boundary conditions, as shown below

(1) $$Q=\frac{\rho }{12\eta }\frac{w}{L}h^{3}P$$

where, *Q* is water flux (g s^−1^), *ρ* is density of water (g cm^−3^) in capillaries, *η* is viscosity of water (Pa s^−1^) in capillaries, *w* is the lateral width (cm) of nanocapillaries, *L* is the permeation length (cm), *h* is the effective pore size (cm), and *P* is the driving pressure (Pa).

Recent studies have shown that this hydrodynamic flow is problematic to describe water transport in some nanocapillaries, e.g., in graphene nanocapillaries, where non‐slip boundary conditions is not applicable. As discussed in Figure 3, due to the weak attraction between water molecules and graphene walls, the friction at the wall is low, giving a non‐zero wall velocity. This “liquid slip” can be quantified by the slip length *δ* ^[^ ^68^ ^]^ (Figure 9a) that increases the hydrodynamic flow velocity by a factor of (1+6*δ*/*h*) and the electro‐osmotic flow velocity by a factor of at least (1+ *δ*/*λ*).^[^ ^69^ ^]^ In the case where only hydrodynamic flow presents, the Hagen‐Poiseuille flow equation can be modified to be as follows^[^ ^25^ ^]^

(2) $$Q=\frac{\rho }{12\eta }\frac{w}{L}h^{3}P\cdot \left(1+\frac{6\delta }{h}\right)$$

where, *δ* is the slip length in cm.

Radha et al.^[^ ^25^ ^]^ used MD simulations and calculated the slip length of water transport in graphene nanocapillaries (<2 nm) to be ≈60 nm, consistent with other simulated results.^[^ ^43^ ^]^ In addition, *P* in Equation (2) was also simulated and found to be highly dependent on *h*. Using these parameters, the relationship between *Q* and *h* was obtained from Equation (2). It was found that, the calculated *Q*(*h*) dependence reproduced the experimental data with striking agreement, and particularly, it predicted the unexpected increase of water flux when *h* < 2 nm.^[^ ^25^ ^]^ Therefore, Equation (2) successfully described water transport behavior in graphene nanocapillaries, where the large enhancement factor of 6*δ*/*h* under severe nanoconfinement (<2 nm) was due to the low friction of water molecules against graphene walls.

#### 2.5.3 Ion Transport in 2D Capillaries

In terms of ion transport in 2D capillaries, ions can be transported with water flow (convection), by an ion concentration gradient (diffusion), or by an electrical potential gradient (migration) (Figure 9b).^[^ ^50b^ ^]^ Taking considerations of all three driving forces, ion flux can be deducted, as described by the classical Nernst‐Planck equation or the PNP equation.^[^ ^70^ ^]^ A modified classical PNP model was used by Cheng et al. to simulate ion diffusion through surface‐charged graphene nanocapillaries, which took into account the ion steric effect in solutions with high concentrations (≈1 m) and under high potentials ($\phi \gg \frac{k_{B}T}{z_{i}e}$).^[^ ^71^ ^]^ The solution for a binary symmetric electrolyte, e.g., KCl, while ignoring the effect of convection, is shown as

(3) $$\frac{\partial n_{i}}{\partial t}=D_{i}\nabla^{2}n_{i}+ez_{i}\mu_{i}n_{i}\nabla \phi +\frac{D_{i}N_{A}a^{3}n_{i}\nabla \left(n_{+}+n_{-}\right)}{1-N_{A}a^{3}\left(n_{+}+n_{-}\right)}$$

where, *a* is the size of cation and anion in KCl aqueous electrolyte, *ϕ* is the electric potential distribution, *ρ* is the net charge density, *N_A_* is the Avogadro constant, *D_i_*, *u_i_*, *z_i_*, *n_i_* are diffusivity, electro‐mobility, valence number, and concentration distribution, respectively, for species *i*. The last term of Equation (3) is the entropy term, which is added as a correction for finite ion size.^[^ ^59^ ^]^

Situations become much more complicated when considering ion transport through severely confined nanocapillaries (<2 nm). Similar to the discussion for Equation (2), the first consideration is the frictional effect of ion‐wall interactions (Figure 9a), which may be treated in the similar way as in Equation (2) by dividing the terms in Equation (3) with “hindrance factors”.^[^ ^72^ ^]^ The second consideration is the effects of ion dehydration and ionic Coulombic blockade. The third consideration is ion–ion correlations when external electrical potential is applied. All these considerations should be included in their terms to generalize the theoretical description of ion transport behavior. Up to now, except for ion–ion correlations,^[^ ^26^ ^]^ other effects have not been incorporated into Equation (3) with experimental justifications. In addition, some factors are not independent, for example, it is highly possible that the degree of ion dehydration would change the frictional effect of ion‐wall interactions. More unusual phenomena may also be discovered in the near future. Therefore, substantial efforts are urgently required in developing theoretical expressions to describe and predict ion transport behavior within various sub‐2‐nm nanocapillaries.

### 2.6 Transport of Organic Dyes through 2D Nanocapillaries

The microscopic understandings of the transport of organic dyes through 2D nanocapillaries have not been as well explored as those of water and ions. There may be two reasons. First, the organic dyes are normally much larger than hydrated ions, and thus, high rejection rates toward the organic dyes are easier to be achieved through size exclusion mechanism, while the size of the nanopores in the NF membranes can be set at a relatively large value that allows fast water/organic solvent flux.^[^ ^73^ ^]^ In addition, for cationic or anionic organic dyes, surface‐charged, 2D material NF membranes can also be adopted to further improve their rejection performance.^[^ ^74^ ^]^ In the literature, the rejection of organic dyes can readily reach over 99%, as will be illustrated in Section 3.^[^ ^2^, ^13^ ^]^ Second, abnormal permeation behavior has yet been discovered so far for the transport of organics dyes through nanocapillaries. On one hand, the dehydration (or desolvation) of the organic dyes may not be a major obstacle in understanding the separation process, also because of their relatively large size as well as their low charge density in the case of charged organic dyes. On the other, due to various forms of organic dyes, their interactions with the membranes, within the nanopores, and among themselves, are rather complicated and difficult to simulate in a convincing way.

**Figure 11:**
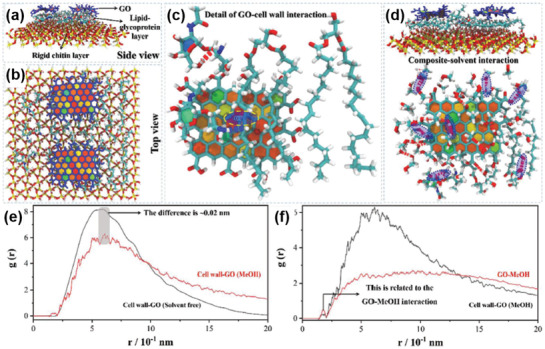
Interaction between fungal cell wall and GO by all atom molecular dynamics. a–c) the configuration and interactions between GO and cell wall; d,e) composite membrane‐solvent interactions. Reproduced with permission.^[^ ^75^ ^]^ Copyright 2021, Wiley‐VCH.

However, progress has been made in recent years. It is noticed that the stability of the NF membranes in water or organic solvents would be critical in sustaining high rejection rates of organic dyes. Moreover, the interactions between the membranes and organic solvents may further render us with indirect hints to understand the rejection of organic dyes. Therefore, before stretching to the transport of organic dyes, the microscopic understandings of the antiswelling property of 2D material NF membranes in organic solvents would be a good starting point for this type of studies. Most recently, Zhang et al.^[^ ^75^ ^]^ attempted this methodology by studying the interactions between a fungal cell wall‐GO (CW‐GO) micro‐composite membrane and organic solvents (methanol, ethanol, etc.), as shown in **Figure** **11**. The atomic model of the micro‐composite membrane was established in Figure 11a,b, where a solid SiO_2_ plate with the molecular fractions of lipids and glycoproteins attached was used to represent the fungal cell wall, on top of which two GO nanosheets were then introduced. Figure 11c showed that both hydrophobic lipid component and hydrophilic glycoproteins component in the cell wall could strongly interact with the amphiphilic GO, through hydrophobic interaction and hydrogen bonding, respectively. The membrane‐solvent interaction was further analyzed in Figure 11d–f. The radial distribution function in Figure 11e showed a small expansion of ≈0.02 nm after adding methanol molecules into the composite membrane model, while the apparent expansion was shown for pure GO membrane mode under the same conditions (Figure 11f).^[^ ^75^ ^]^ Hence, it illustrated that the cell wall could not only stabilize the GO sheets by directly interacting with them, but also restrict the expansion of the composite layers. This explained the excellent antiswelling property of this composite membrane, superior to that of pure GO membranes, when rejecting organic dyes (Evans Blue) from various organic solvents.^[^ ^75^ ^]^ This work may also suggest that strong interactions between the membrane and organic dyes are not favored for the effective rejection. The strong interactions may induce the dissociation or significant expansion of the membrane structure during the separation process; in addition, it may also cause the blockage of the nanopores after a long‐term operation. Inspired by this understanding, the simulation of the strength of the interactions between the membrane and organic dyes could provide us with a reasonable guideline of selecting suitable membrane structural designs for the separation.

## 3 State‐of‐the‐Art Designs of 2D Material NF Membranes

According to the above‐mentioned fundamental understandings, fine tuning of pore structures, including pore size, pore chemistry, and pore geometry, in 2D material NF membranes is critical in achieving fast water flux and high rejection rates. Hence, in reviewing the recent membrane structural designs, we will focus on the newly developed measures that have been developed to precise control the interlayer spacing of the restacked 2D material NF membranes. In addition, the recent designs for the structural control of single‐layer 2D material NF membranes, MOF‐2D material composite NF membranes, and surface‐charged GO membranes are also included in this review. Although many 2D materials have been attempted to construct membranes, here we mainly focus on GO, rGO, and MXenes nanosheets, which are the most studied 2D materials in this area.^[^ ^76^ ^]^ A detailed review of 2D materials membranes for critical separations can be seen in the report by Liu et al.^[^ ^13a^ ^]^ Furthermore, when comparing the performance of water flux and rejections rates among various studies, the experimental method and some key experimental descriptions should be provided, as applied in the discussion in the following sections. Special attention should also be given to the differences between the rejection of ions and the rejection of organic dyes since the rejection of ions is fundamentally more difficult than that of organic dyes. Antiswelling property is another key factor when evaluating the overall performance of membranes for water purification.

### 3.1 Precise Interlayer Spacing Control of Restacked 2D Material NF Membranes

In the restacked 2D material NF membranes, pore geometry is 2D slit pores, pore chemistry relies on the nature and the surface chemistry of 2D materials, pore size is determined by the restacked interlayer spacing, and strictly speaking, the free space of *d*‐spacing; and thus, developing methods that can precisely modulate the restacked interlayer spacing between those 2D materials is technically vital. Recent progress on the designs of manipulating *d*‐spacing mainly include the following three categories, cationic control, covalent bridging, and multilayer build‐up.

#### 3.1.1 Cationic Control of the Restacked Interlayer Spacing

**Figure 12:**
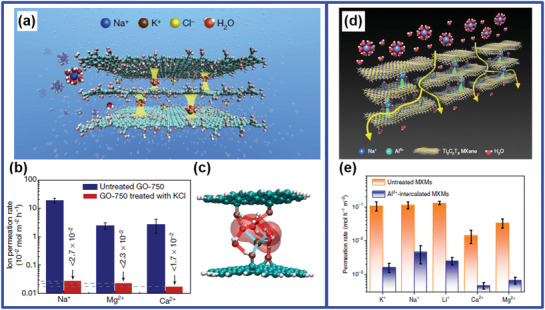
Cationic control. a–c) KCl‐controlled GO membranes: a) a schematic of how K^+^ ions in a GO membrane determine and fix the interlayer spacing such that other cations are rejected while pure water can penetrate; b) Na^+^, Ca^2+^, and Mg^2+^ permeation rates of untreated and KCl‐controlled GO membranes; c) the most stable optimized geometry of K^+^‐(H_2_O)_6_@GO clusters from DFT computation. a‐c) Reproduced with permission.^[^ ^78^ ^]^ Copyright 2017, Springer Nature. d,e) Al^3+^‐intercalated MXene membranes: d) schematic of how Al^3+^ intercalates the two adjacent MXene layers and thus fixes the *d*‐spacing; e) ion permeation rates through untreated MXMs and Al^3+^‐intercalated MXMs. d,e) Reproduced with permission.^[^ ^81^ ^]^ Copyright 2020, Springer Nature.

Inspired by alkali‐metal cation intercalation in layered materials in batteries, such as lithium‐ion batteries and sodium‐ion batteries, cationic control of interlayer spacing has been demonstrated to be a promising membrane structural design with angstrom precision.^[^ ^77^ ^]^ Cheng et al.^[^ ^78^ ^]^ reported that, by immersing a GO membrane into KCl solution, hydrated K^+^ in the solution could be inserted into the restacked GO nanosheets and fixed the interlayer spacing of the GO membrane to be ≈11.4 Å (**Figure** **12**a). In this way, the permeability of other cations, e.g., Na^+^, Mg^2+^, and Ca^2+^, had been substantially reduced and some to be undetectable by inductively coupled plasma optical emission spectrometer (ICP‐OES) (Figure 12b).^[^ ^78^ ^]^ This was attributed to the size exclusion mechanism because the size of the hydrated K^+^ is the smallest among these four cations. Importantly, the antiswelling properties had also been improved, suggesting the strengthened interactions within 2D GO nanocapillaries after the insertion of hydrated K^+^.^[^ ^78^ ^]^ Using density functional theory (DFT) computation (Figure 12c), it was suggested that the fixing of the interlayer spacing was mainly due to the interaction between the hydrated K^+^ and aromatic rings on the GO nanosheet (cation‐*π* interactions^[^ ^79^ ^]^), as well as the interaction between the hydrated K^+^ and the oxidized groups on the GO nanosheet. It was also noted that hydrated K^+^ fixed the interlayer spacing better than all other tested hydrated cations.^[^ ^78^ ^]^ Based on the microscopic understandings discussed in Section 2, we suspect that there was reconfiguration of the hydration shell of K^+^ after its insertion, which partially exposed the inner core of K^+^ and strengthened the interactions. Since the dehydration energy barrier is the lowest for hydrated K^+^ compared with other hydrated cations, hydrated K^+^ showed the best performance in this work. This understanding may be illustrated in the simulated result in Figure 12c as well.

Although Cheng et al. had demonstrated superior ion rejection rates by the insertion of hydrated K^+^ into GO membranes, the water flux presented was as low as 0.1 L m^−2^ h^−1^ obtained at their best ion permeation performance in FO test (U‐shape cell).^[^ ^78^ ^]^ It is also understandable because the oxygenated functional groups present in 2D nanocapillaries can significantly retard water transport. In other studies, fast water flux had been reported for MXene‐based NF membranes,^[^ ^80^ ^]^ and hence, Ding et al. inserted Al^3+^ cations into the interlayer of Ti_3_C_2_‐MXene nanosheets in the membranes (Figure 12d). Significantly improved ion rejection performance (Figure 12e) was achieved for the membranes with good antiswelling properties.^[^ ^81^ ^]^ At the same time, a mediate water flux of 1.1 L m^−2^ h^−1^ at the high ion rejection of 99.6% NaCl and a high water flux of 8.5 L m^−2^ h^−1^ at the mediate ion rejection of 89.5% NaCl were achieved based on the Al^3+^‐intercalated MXene membranes using FO, U‐shape cell.^[^ ^81^ ^]^ It is noted that the surface of Ti_3_C_2_‐MXene nanosheets is also covered with O terminated groups, and thus, water transport should also be impeded in the 2D MXene nanocapillaries as that in 2D GO nanocapillaries. The underlying reason of the relatively fast water flux obtained on MXene‐based membranes is still unknown. Furthermore, although the modulation of the interlayer spacing of MXene nanosheets by cation intercalation has been reported in many papers,^[^ ^61^, ^82^ ^]^ their application as NF membranes in water purification is still struggling, maybe restricted by the instability of MXene nanosheets in aqueous solutions.^[^ ^83^ ^]^

The most competitive advantage for the design of cationic control is its ability to manipulate the interlayer spacing with angstrom precision. Obstacles, however, also exist. For example, the hydration layer of the inserted cations can substantially reduce the interaction between the cations and the capillary wall; and more challenging is, this interaction normally requires the functionalization of capillary walls, which may sacrifice the water flux. Therefore, it is extremely interesting to see whether this method can be applied onto the hydrophobic 2D nanocapillaries, e.g., pristine graphene nanocapillaries (<2 nm), for the simultaneous improvement of water flux as well as ion rejection rates.

#### 3.1.2 Covalent Bridging by Small Organic Molecules

**Figure 13:**
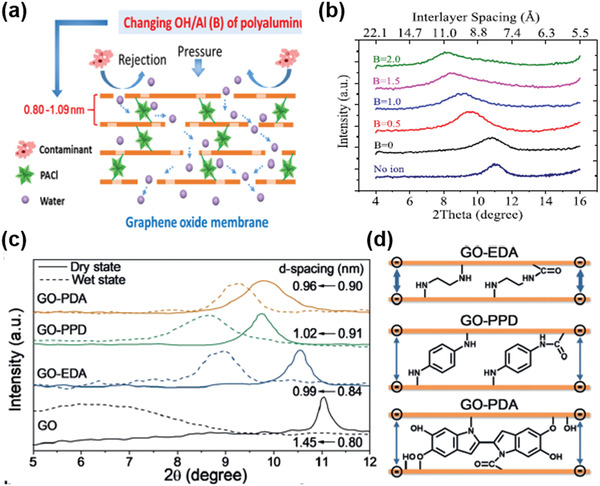
Covalent bridging. a) A schematic of GO membranes with adjustable interlayer spacing and high stability in aqueous solutions through cross‐linking with polyaluminum chloride (PACl). b) XRD spectra for different PACl‐GO membranes with 1 × 10^−3^ and 10 × 10^−3^ m PACl. a,b) Reproduced with permission.^[^ ^82^ ^]^ Copyright 2019, American Chemical Society. c) The antiswelling capabilities of interlaminar short‐chain molecularly bridged GO membranes: XRD patterns (left) of pristine GO and EDA‐, PPD‐, and PDA‐bridged GO membranes. d) An illustrations (right) of GO‐EDA, GO‐PPD, and GO‐PDA laminates. c,d) Reproduced with permission.^[^ ^86^ ^]^ Copyright 2020, Wiley‐VCH.

Covalently bridging adjacent GO nanosheets, e.g., using small organic molecules as cross‐linkers, is an effective design to tune interlayer spacing as well as to prevent membranes from swelling.^[^ ^84^ ^]^ 1,3,5‐benzenetricarbonyl trichloride (TMC) was used to cross‐link adjacent GO nanosheets by a layer‐by‐layer (LBL) deposition. High water flux had been obtained (8–27.6 L m^−2^ h^−1^ bar^−1^) in dead‐end membrane filtration system but with low salt rejection rates (6–46%).^[^ ^85^ ^]^ Polyaluminum chloride (PACl) was attempted to be intercalated into the GO nanosheets forming strong binding between highly charged Al_13_ polymers (Al_13_O_4_(OH)_24_^7+^) and negatively charged GO nanosheets (**Figure** **13**a).^[^ ^82^ ^]^ Interlayer spacing could be tuned between 8.0 and 10.9 Å (Figure 13b). In this way, high water flux of ≈80 L m^−2^ h^−1^ bar^−1^ could be obtained under vacuum filtration test at 1 bar with acceptable rejection rates for organic dyes (> 90%), but no data were reported for ion rejection rates in the study.^[^ ^82^ ^]^ Recently, Zhang et al. used ethylenediamine (EDA), paraphynylenediamine (PPD), and polydopamine (PDA) individually to form short‐chain covalent bridges between adjacent GO nanosheets (Figure 13c,d), and meanwhile, used aldehyde (glutaraldehyde or maleic anhydride)‐modified chitosan as an interfacial long‐chain molecular bridge between the GO membrane and the porous substrate.^[^ ^86^ ^]^ Surprisingly, it was demonstrated that the modified GO membrane could sustain high‐power sonication for 30 min in water.^[^ ^86^ ^]^ A high water flux of 8.1 L m^−2^ h^−1^ bar^−1^ could be obtained under cross‐flow, high pressure, and continuous process, with a high rejection rate for Rhodamine B (RhB) (98.3%).^[^ ^86^ ^]^ Nevertheless, no ion rejection rates were reported in the study.

Although this is an interesting method in controlling the interlayer spacing with good precision and stable structure, this method has relatively low rejection rates to monovalent and divalent ions. One exception is that, Zhang et al.^[^ ^84d^ ^]^ used EDA to cross‐link GO nanosheets which was then covered with a layer of hyperbranched polyethyleimine (HPEI) on the surface. The amine‐modified GO membrane showed high water flux of 5.01 L m^−2^ h^−1^ bar^−1^ in dead‐end permeation test and relatively high rejection toward heavy metal ions (90–98%), such as Mg^2+^, Pb^2+^, Cd^2+^, and Zn^2+^.^[^ ^84d^ ^]^ Despite the progress, substantial efforts are needed in this direction. As indicated in the discussion of the microscopic understandings in Section 2, *d*‐spacing of GO membranes smaller than 10 Å (Figure 13b,c) should be good enough to exclude hydrated monovalent ions, e.g., hydrated K^+^, Na^+^ or Li^+^ cations, however, this was not the case for the method of covalent bridging by small molecules. The underlying reason requires further investigation. In addition, how can these organic cross‐linkers affect water transport? Would these organic cross‐linkers change their configurations in aqueous solution or during the ion/water transport processes? The understandings of these questions would provide us with a guidance in choosing or designing suitable cross‐linkers to further improve the overall performance of the NF membranes for water purification.

#### 3.1.3 Building Multilayer Architectures

Both cationic control and covalent bridging require the presence of functional groups on 2D nanocapillary walls in assisting the formation of new bonding to connect the adjacent layers, such as the oxygenated functional groups on GO or O terminations on MXene nanosheets. This feature of pore chemistry results in a general trend, that is, small slit pores would be effective in improving ion rejection but deteriorating water flux, while relatively large slit pores would enhance water flux but worsen ion rejection performance although the rejection rates for organic dyes is sometimes acceptable, as shown in Figures 12 and 13. 2D graphene nanocapillaries (<2 nm) with a large capillary pressure can solve this dilemma by improving water flux and ion rejection rates simultaneously, as discussed in Section 2; nevertheless, the restacking of rGO nanosheets is always a problem. Therefore, build‐up of hydrophilic/hydrophobic multilayered membranes may be a promising solution.^[^ ^61^, ^74^, ^87^ ^]^

Previous attempts first studied the fabrication of ultrathin rGO membranes for water purification. Han et al.^[^ ^38a^ ^]^ reported the use of ultrathin rGO membranes (22–53 nm) for the nanofiltration and as high as 21.8 L m^−2^ h^−1^ bar^−1^ water flux was obtained using dead‐end filtration system (applied pressure of 5 bar). Although over 99% of rejection rates were achieved for organic dyes, it exhibited moderate ion rejection rates (20–60%).^[^ ^38a^ ^]^ Liu et al. reported the fabrication of 100 nm freestanding rGO membranes via hydrogen iodide (HI) steam as the reducing agent.^[^ ^88^ ^]^ In cross‐flow FO test with no external pressure, the partially reduced rGO membrane presented a high water flux of 57 L m^−2^ h^−1^, but still, a moderate ion rejection rate of 0.2 mol h^−1^ m^−2^ was obtained for Na^+[^ ^88^ ^]^ when comparing with 2.7 × 10^−4^ mol h^−1^ m^−2^ for KCl‐controlled GO membranes in Figure 12b. It is believed that hydrophobic nanopores could be formed within rGO membranes after partial reduction, which leads to fast water flux originated from the large capillary pressure within these highly confined 2D graphene nanocapillaries. However, XRD test could not pick up any peaks for the nanopores,^[^ ^88^ ^]^ suggesting that the nanopore size may have a wide distribution causing the ion rejection rate being not as high as that for KCl‐controlled GO membranes (Figure 12b).

**Figure 14:**
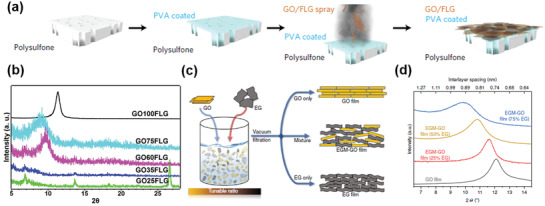
Multilayer architectures. a) Spray coating process of hybrid GO/FLG/deoxycholate layered membranes on PVA‐coated polysulfone substrates. b) XRD patterns of GO/FLG/deoxycholate membranes with the GO content of 100% (GO100FLG), 75% (GO75FLG), 60% (GO60FLG), 35% (GO35FLG), and 25% (GO25FLG). a,b) Reproduced with permission.^[^ ^89^ ^]^ Copyright 2017, Springer Nature. c,d) EG‐GO films with tunable interlayer spacing: c) schematic diagram illustrating the production of EG‐GO films from an aqueous dispersion with a tunable precursor ratio of GO to EG; d) XRD patterns of the as‐prepared EG‐GO films, showing that the interlayer distance is controllable with the relative weight content of EG. c,d) Reproduced with permission.^[^ ^90^ ^]^ Copyright 2020, Springer Nature.

In this sense, the design of GO/rGO multilayer architectures has been proposed. Gomez et al. reported a spray coating method to fabricate hybrid GO/few‐layered graphene (FLG)/deoxycholate layered membranes on polyvinyl alcohol (PVA)‐coated polysulfone substrates (**Figure** **14**a).^[^ ^89^ ^]^ It was demonstrated that the multilayered membranes were robust enough to withstand strong cross‐flow shear for 120 h while maintaining NaCl rejection near 85% at a high water flux of 18 L m^−2^ h^−1^ (0.36 L m^−2^ h^−1^ bar^−1^ by cross‐flow filtration system under the pressure of 5 MPa).^[^ ^89^ ^]^ The best combination of the performance was achieved with the GO content in the multilayered membranes being in the range from 40% to 60%. The corresponding shift of XRD peaks can be seen in Figure 14b.^[^ ^89^ ^]^ The fine tuning of the interlayer spacing of GO/rGO multilayer architectures was also achieved in other studies, e.g., mixing different ratios of GO and exfoliated graphene (EG) (Figure 14c,d).^[^ ^90^ ^]^ The build‐up of GO/MXene multilayer architectures has also been studied, the details of which can be seen in a recent review regarding the applications of MXene‐based membranes for water purification.^[^ ^76b^ ^]^

The ratio of hydrophobic channels can be substantially increased in hydrophilic/hydrophobic multilayered membranes, together with the tunable interlayer spacing and relatively strong binding. Hence, the enhancement of the overall performance can be projected according to the mechanistic understanding discussed in Section 2. This is an attractive direction because it avoids the difficulties of fabricating the membranes with pure hydrophobic graphene nanocapillaries (<2 nm). In addition, this method also renders us with versatile tools in modulating slit pore size as well as pore chemistry.

In short, cationic control has been proven to manipulate the interlayer spacing with angstrom precision in achieving near complete rejection of various hydrated ions. However, the interlayer spacing cannot be fixed with sufficient strength and the water flux would also be suppressed. Covalently bridging, on the other hand, is an effective design to tune interlayer spacing as well as to fix the interlayer spacing strongly; however, this method has relatively low rejection rates to monovalent and divalent ions with the underlying reasons ready to discover. Build‐up of hydrophilic/hydrophobic multilayered membranes can improve water flux and ion rejection rates simultaneously, by partially preventing the restacking of hydrophobic nanosheets. Nevertheless, it has not taken full advantage of 2D hydrophobic nanocapillaries (<2 nm). All three designs are crucial and promising for the precise interlayer spacing control of the restacked 2D material NF membranes, although all of them require further development in the future.

### 3.2 Recent Advances of Other Structural Modulation Designs for 2D Material NF Membranes

#### 3.2.1 New Designs on Single‐Layer 2D Material NF Membranes

In addition to the precise control of slit pore structures in the restacked 2D material NF membranes, the control of pore structures on single‐layer 2D material NF membranes is also interesting since the length of their permeation pathways is exceptionally short, normally smaller than 2 nm if considering the thickness of the hydration layer on the surface of 2D materials.^[^ ^55b^ ^]^ In this case, pore geometry is cylindrical in shape, pore size is the opening size of the cylindrical pores, and pore chemistry is the chemical functionalization of the cylindrical pore surface that is determined by pore‐forming methods. Simulation work had been conducted by creating hydrogenated and hydroxylated nanopores in a single‐layer graphene.^[^ ^91^ ^]^ It was shown that the salt rejection capability decreased with an increasing pore size and the critical pore size was 5.5 Å in diameter for the complete rejection of Na^+^ and Cl^−^ ions from water. In addition, hydroxylated pores had higher water flux than that of hydrogenated pores of the same size, which could be attributed to the electrostatic interactions between the pores and ions.^[^ ^91^ ^]^

**Figure 15:**
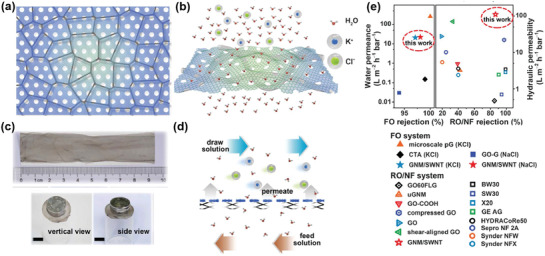
Single‐layer 2D material NF Membranes. a) Designed structural model of the GNM/SWNT hybrid membrane with single‐layer GNM supported on SWNT networks. b) Structural model of the GNM/SWNT hybrid membrane for size exclusion nanofiltration. c) Photograph of the large area GNM/SWNT membrane and those of the GNM/SWNT hybrid membrane suspended on a tube with six coins on the membrane. d) Sketch of water permeance through the GNM/SWNT membrane driven by osmotic pressure in the cross‐flow FO system. e) Comparison of the water‐permeability and salt‐rejection performance of the GNM/SWNT hybrid membranes with commercial osmosis membranes and graphene‐based separation membranes. Reproduced with permission.^[^ ^92^ ^]^ Copyright 2019, AAAS.

For the experimental work, O'Hern et al. introduced reactive defects into the graphene lattice using ion bombardment followed by oxidation etching.^[^ ^23b^ ^]^ Later on, Surwade et al. reported an oxygen plasma etching process to create nanoscale pores in a single‐layer graphene. Nearly 100% ion rejection had been achieved for this membrane with rapid water transport of 10^6^ g m^−2^ s^−1^ (3.6 × 10^6^ L m^−2^ h^−1^ under the transmembrane pressure difference only ≈17 kPa).^[^ ^93^ ^]^ Similar methods have also been reported to generate nanopores on a single layer MoS_2_.^[^ ^23a^ ^]^ Although superior performance had been reported on single‐layer 2D material NF Membranes, it was strictly limited to proof‐of‐concept demonstrations on micrometer‐scale graphene nanosheets (10^−6^–10^−8^ cm^2^).^[^ ^92^ ^]^ Recently, Yang et al. reported an attractive membrane structure, a large‐area graphene nanomesh (GNM) on a single‐walled CNT (SWNT) network, forming a GNM/SWNT hybrid membrane (**Figure** **15**a,b).^[^ ^92^ ^]^ This hybrid membrane structure had excellent mechanical strength (Figure 15c), because the interconnected SWNT web featured a strong *π*‐*π* interaction with the supported GNM and physically separated the GNM into microsized islands. Using a cross‐flow filtration setup (Figure 15d), the GNM/SWNT hybrid membrane showed a high water flux of 37.2 L m^−2^ h^−1^ bar^−1^ for a high ion rejection rate of 98.1% NaCl in cross‐flow FO test, and a high water flux of 97.6 L m^−2^ h^−1^ bar^−1^ for a high ion rejection rate of ≈90% NaCl in cross‐flow RO test (Figure 15e).^[^ ^92^ ^]^ With this significant advance, the ease of membrane fabrication using a cost‐effective manner may be the next target for this direction. This work may also provide us with a diversified platform to study the combination effects of cylindrical pore size and pore chemistry on ion dehydration and ionic Coulombic blockade processes, which should be critical in understanding water/ion transport behavior in membrane technologies as well as ion storage behavior in nanopores for energy storage fields. Antifouling property of this type of membranes may be a concern, which requires further studies in the future.

#### 3.2.2 New Designs on MOF‐2D Material Composite NF Membranes

The composite membranes, such as GO‐polymer,^[^ ^74^ ^]^ GO/rGO‐metal oxide,^[^ ^94^ ^]^ and GO/rGO‐CNT composite membranes,^[^ ^10^, ^95^ ^]^ use a second material as a spacer to expand the slit pore size of 2D material NF membranes. The *d*‐spacing, in this case, cannot be controlled in a precise way. MOF‐2D material composite NF membranes, on the other hand, is a special case because the spacer made of MOFs possesses highly uniform and angstrom‐precise pore structures.^[^ ^96^ ^]^ Pang et al.^[^ ^97^ ^]^ reported nanosized UiO‐66/GO sandwiched membranes for water purification, where the hydrophilic MOF nanoparticles worked as the microporous spacer. UiO‐66 has a high porosity of ≈1200 m^2^ g^−1^, a narrow and uniform pore size of 6.0 Å, suitable for preventing hydrated Na^+^ transport and reversed draw solute diffusion.^[^ ^98^ ^]^ It was demonstrated that UiO‐66/GO membrane exhibited a fast water flux of 29.16 L m^−2^ h^−1^ in cross‐flow FO test under the applied pressure of 1 bar, which was 270% higher than the pure GO membranes, while the reverse solute (NaCl) diffusion was reduced by 83.5%.^[^ ^97^ ^]^ The application of MOF‐based membranes in water purification can be seen in a recent review.^[^ ^99^ ^]^

**Figure 16:**
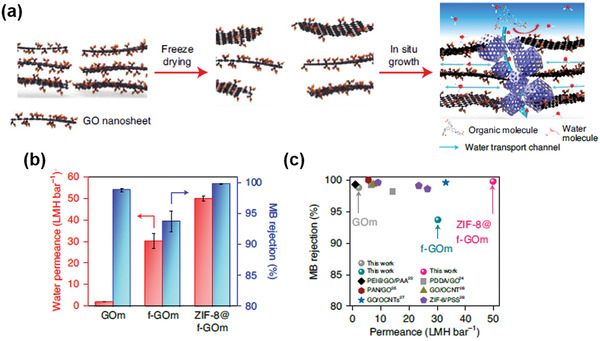
MOF‐2D material composite NF Membranes. a) Schematic of ZIF‐8@freeze‐dried GO membranes (ZIF‐8@f‐GOm) preparation, in which the GO framework is freeze dried by the ice‐templating technique before subsequently growing ZIF‐8 nanocrystals in the microporous defects to act as mechanical supports. b) Water permeance and MB rejection for GO‐based membranes. c) Comparison of MB rejection and water permeance for GO‐based membranes under cross‐flow conditions. Reproduced with permission.^[^ ^73^ ^]^ Copyright 2021, Springer Nature.

More recently, Zhang et al. reported an interesting method to composite MOF crystals with GO membranes.^[^ ^73^ ^]^ Instead of using MOFs only as the microporous spacers, zeolitic imidazolate framework‐8 (ZIF‐8) nanocrystals were in‐situ crystallized at GO nanosheet edges to seal the relatively large interedge pores and replace them with the angstrom‐sized pores in MOFs (**Figure** **16**a). This particular composite membrane structure not only optimized the interedge pores, but also enlarged the interlayer spacing, imparted mechanical integrity to the laminate framework, and thus, produced a stable microstructure capable of delivering a high water flux of 60 L m^−2^ h^−1^ bar^−1^ (cross‐flow filtration system under the applied pressure of 1 bar) for 180 h and near‐perfect methylene blue (MB) rejection of ≈100% (Figure 16b,c).^[^ ^73^ ^]^ However, the ion rejection rates reported for this composite membrane were limited to be lower than 60%, and only around 30% for the rejection of NaCl. It is noted that, although MOF materials are microporous with highly regulated pore sizes, their 3D particulate in nature still causes difficulties in controlling the interlayer spacing with angstrom precision. Thus, we expect that 2D MOF materials^[^ ^100^ ^]^ may take an important role in developing MOF‐2D material composite NF membranes with fast water flux, high ion rejection rates, and good antiswelling properties.

#### 3.2.3 The Design of Surface‐Charged GO Membranes

**Figure 17:**
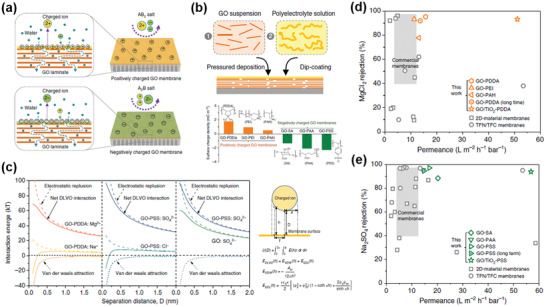
Surface‐charged GO membranes. a) Schematic of the design of surface‐charged GO membranes by coating polyelectrolytes on the surface of GO laminates to realize controllable ion transport. b) Schematic of the preparation of surface‐charged GO membranes and surface charge densities calculated from the measured membrane zeta potentials. c) Surface element integration model predictions of DLVO interaction energies between a charged ion and the charged membrane surface. d) MgCl_2_ rejection with water permeance of positively charged GO membranes and e) Na_2_SO_4_ rejection with water permeance of negatively charged GO membranes obtained in this work, as well as the comparison with other 2D material membranes and commercial polymeric nanofiltration membranes. Reproduced with permission.^[^ ^102^ ^]^ Copyright 2019, Springer Nature.

Surface charge on 2D material composite NF membranes is able to exclude ions or charged organic dyes by the charge interaction principle.^[^ ^2^, ^80^, ^101^ ^]^ Recently, Zhang et al. created surface charges on GO membranes and realized controllable ion transport without impeding water permeation (**Figure** **17**a).^[^ ^102^ ^]^ Coating polycations, such as polydiallyl dimethyl ammonium (PDDA), polyethylene imine (PEI), and polyallylamine hydrochloride (PAH) let a GO membrane to exclude AB_2_‐type salts, while coating polyanions, such as polystyrene sulfonate (PSS), polyacrylic acid (PAA), and sodium alginate (SA) led the GO membrane to exclude A_2_B‐type salts, as illustrated in Figure 17b. Using DLVO theory, it was found that the charged membrane surface exhibited a much higher energy barrier for high‐valent co‐ion transport than that for the low‐valent co‐ion transport (Figure 17c). Therefore, for MgCl_2_, a positively charged membrane would exhibit a dominant electrostatic repulsion against Mg^2+^ than in attraction to Cl^−^. To balance the charge in solution, Cl^−^ counter‐ions would be excluded simultaneously. Similar case for Na_2_SO_4_ on negatively charged membranes.^[^ ^102^ ^]^ Further by intercalating nanoparticles into the surface‐charged GO membranes, water permeation could be substantially enhanced at almost no cost of ion rejection. It was shown that the rationally designed surface‐charged GO membranes exhibited MgCl_2_ rejection of 93.2% with a fast water flux of 51.2 L m^−2^ h^−1^ bar^−1^ and Na_2_SO_4_ rejection of 93.9% with a fast water flux of 56.8 L m^−2^ h^−1^ bar^−1^, tested under dead‐end filtration system under the applied pressure of 2 bar (Figure 17d,e).^[^ ^102^ ^]^

Although this method is applicable only to the system with multivalent ions, it is interesting to see that the ion exclusion through charge interaction principle can be well coupled with the composite membrane design, by taking advantage of both designs while avoiding their individual drawbacks. How to integrate size exclusion mechanism with like‐charge exclusion mechanism to reject ions effectively while preserving high performance in other aspects, such as water flux, antiswelling, antifouling, mechanical properties, etc., is always a promising direction for the novel designs of membrane technologies in water purification.

## 4 Outlook

### 4.1 Some Basic Guidelines and Precautions of Designing 2D Material NF Membrane toward Commercialization

Despite the fast development of 2D material NF membranes, concerns of applying these membranes in water treatment applications, ranging from seawater desalination, water reuse and other industrial water treatment, have been raised by many researchers and experts.^[^ ^103^ ^]^ It was claimed that current commercial RO membranes made by thin‐film‐composite (TFC) polyamide offer a greater than 10‐fold reduction in specific energy consumption (SEC) compared to thermal technologies (e.g., distillation).^[^ ^104^ ^]^ Further increasing the water flux above the values of TFC membranes offers little opportunity for reducing SEC especially in seawater RO.^[^ ^103b^ ^]^ From this perspective, high water flux alone if achieved on 2D material NF membranes cannot be translated to apparent energy saving in water‐treatment system level. In addition, high ion rejection rate weights more significantly than the magnitude of water flux in practical use, and next‐generation membranes with ultra‐high rejection could obviate the need for extensive post‐treatment steps, such as additional RO passes, saving cost and energy.^[^ ^103b^ ^]^ Therefore, the ongoing efforts for the design of novel 2D material NF membranes should be focused on achieving high water flux and high ion rejection rate at an externally applied pressure as low as possible, with emphasis put more on the latter.

Besides the extent of energy saving, fabrication cost and scalability are also two critical obstacles impeding 2D material NF membranes toward commercialization. In terms of fabrication cost, GO‐based membranes may be the best option among all 2D material NF membranes after over a decade of development of GO in academic field and in industries. It is noted that membrane fouling is highly correlated to the cost of the membranes from the operation point of view. Moreover, the intrinsic defects are inevitable in the restacked 2D material NF membranes,^[^ ^103c^ ^]^ and the mechanical property of the single‐layer and the restacked 2D material NF membranes is always a concern when scaling up. Fortunately, more and more papers started to address these issues and demonstrate the scalability of the membranes in their work, at least to some extent.^[^ ^74^, ^86^, ^92^ ^]^ Hence, the evaluation of future 2D material NF membranes is suggested to be conducted comprehensively within the practical performance metrics, that is, permeation performance (water flux, ion rejection, and antiswelling), SEC, cost, and scalability, and in direction comparison to current commercial TFC membranes using a holistic and convincing manner. New designs of novel 2D material NF membranes should therefore not be focused on the enhancement of one or two particular performance, but the overall performance as a whole. Balance of the overall performance may be reached differently for specific cases in water treatment applications.

Note that there exists some inappropriate interpretation of physical phenomena^[^ ^105^ ^]^ as well as improper performance calculations^[^ ^106^ ^]^ in the literature. Precautions should be taken when evaluating true performance of the membranes.

### 4.2 Conclusion and Perspectives on the Rational Design of 2D Material NF Membranes

**Figure 18:**
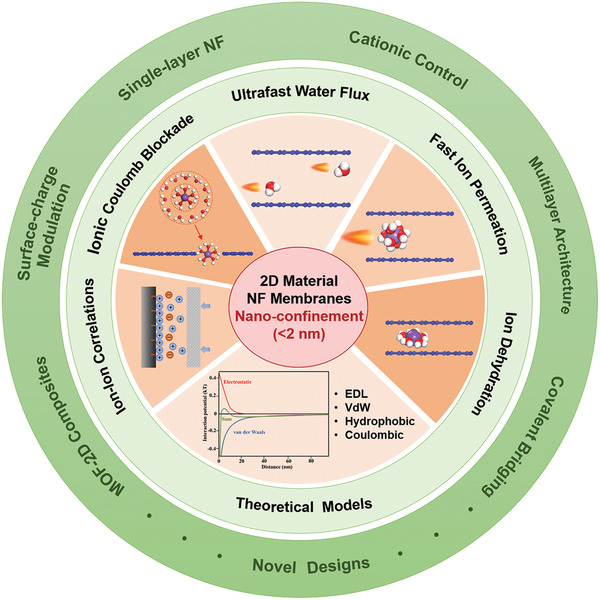
Schematic of water/ion transport behaviors through highly confined, sub‐2‐nm capillaries and the state‐of‐the‐art designs of the 2D material NF membranes, guiding the novel designs in the future.

In spite of the existing challenges toward commercialization, 2D material NF membranes have demonstrated great potential to be an energy‐saving and cost‐effective manner in achieving high performance for water purification, including high water flux, high rejection rates of ions, high resistance to swelling, etc. The potential arises from the abnormal phenomena of water and ion permeation through highly confined sub‐2‐nm capillaries. The water/ion transport behaviors and the state‐of‐the‐art designs of the 2D material NF membranes have been concluded in **Figure** **18**, and their water purification performance has also been summarized in **Table** **1**.

It was found that water transport within 2D graphene nanocapillaries (<2 nm) is anomalously fast with the density of water intercalated being higher than that of bulk water, which can be attributed to the large capillary pressure (≈1000 bar) originated from van der Waals attraction between water molecules and graphene walls. Oxygenated functional groups on GO membranes, on the other hand, would impede water flux. These understandings suggest that, by constructing the restacked 2D material NF membranes possessing 2D hydrophobic graphene nanocapillaries (<2 nm) with the least amount of oxygenated functional groups present, high water flux can be realized at low externally applied pressures, and meanwhile, high ion rejection rates can also be achieved through size exclusion mechanism. However, technically, this membrane structural design has not been acquired yet due to the easy restacking of rGO nanosheets. The design of multilayered membranes is a compromised method and the design of using single‐layer 2D material NF membranes can circumvent this strict requirement by reducing the permeation length down to sub‐2 nm. Other designs, such as cationic control, covalent bridging, and MOF‐2D material composite, have not been attempted to incorporate this unusual enhancement of water flux. Therefore, substantial research efforts are expected in the future to directly achieve this targeted membrane structural design. More importantly, fine tuning of the slit pore size of the 2D hydrophobic graphene nanocapillaries with angstrom precision, using the way that can also render the membranes with good antiswelling property, should be developed.

**Table 1:** Summary of water flux, ion rejection, and/or dye rejection performance for the state‐of‐the‐art designs of 2D material NF membranes

2D Material NF Membrane Design	Thickness	Driving Force	Testing Method	Feed Solution	Water Flux [L m^−2^ h^−1^]	Water Permeance [L m^−2^ h^−1^ bar^−1^]	Ion^a)^ Rejection	Dye Rejection	Ref.
GO Membrane	750 nm	Osmotic Pressure	FO U‐shape cell	0.25 m NaCl	0.17	–	0.18 mol m^−2^ h^−1^	–	^[^ ^78^ ^]^
KCl‐controlled GO	750 nm	Osmotic Pressure	FO U‐shape cell	0.25 m NaCl	0.1	–	2.7 × 10^−4^ mol m^−2^ h^−1^	–	^[^ ^78^ ^]^
Shear aligned GO Membrane	220 nm	External Pressure (0.5 bar)	Dead‐end cell	–	20	40	–	≈98% MB^b)^	^[^ ^76d^ ^]^
Shear aligned GO Membrane	≈150 nm	External Pressure (0.5 bar)	RO Dead‐end cell	0.034 m NaCl	35.5	71	25–40%	–	^[^ ^76d^ ^]^
MXene Membrane	1.5 µm	–	Dead‐end Filtration	0.2 m NaCl	–	37.4	–	–	^[^ ^80^ ^]^
MXene Membrane	1.5 µm	Osmotic Pressure	FO U‐shape cell	0.2 m NaCl	–	–	1 mol m^−2^ h^−1^	–	^[^ ^80^ ^]^
Al^3+^‐intercalated MXene Membranes	1.1 µm	Osmotic Pressure	FO U‐shape cell	0.1 m NaCl	–	–	4 × 10^−3^ mol m^−2^ h^−1^	–	^[^ ^81^ ^]^
Al^3+^‐intercalated MXene Membranes	2.7 µm	Osmotic Pressure (2 m sucrose)	FO U‐shape cell	0.1 m NaCl	1.1	–	99.6%	–	^[^ ^81^ ^]^
Al^3+^‐intercalated MXene Membranes	340 nm	Osmotic Pressure (2 m sucrose)	FO U‐shape cell	0.1 m NaCl	8.5	–	89.5%	–	^[^ ^81^ ^]^
MXene‐derived Membrane	≈100 nm	External Pressure (3 bar)	RO Cross‐flow test	0.1 m NaCl	18	6	55.3%	–	^[^ ^82^ ^]^
GO/Polycation	≈100 nm	External Pressure (5 bar)	RO Cross‐flow test	0.1 g L^−1^	17.2	3.44	–	99.3% MB	^[^ ^84a^ ^]^
GO/TMC	≈25 nm	External Pressure (3.4 bar)	Dead‐end cell	Pure water	93.8	27.6	–	–	^[^ ^85^ ^]^
GO/TMC	≈25 nm	External Pressure (3.4 bar)	Dead‐end cell	0.02 m NaCl	–	–	≈20%	–	^[^ ^85^ ^]^
GO/TMC	≈25 nm	External Pressure (3.4 bar)	Dead‐end cell	7.5 mg L^−1^ MB	–	–	–	≈62% MB	^[^ ^85^ ^]^
GO/PACl	273 nm	External Pressure (1 bar)	Pressurize Filtration	10 mg L^−1^ SA^c)^	105	105	–	95% SA	^[^ ^82^ ^]^
GO‐PDA/O = CS/ceramic Membrane	80 nm	External Pressure (1–10 bar)	RO Cross‐flow test	–	–	4.3–8.1	–	> 98% RhB	^[^ ^86^ ^]^
HPEI‐modified GO/EDA Membrane^d)^	70 nm	External Pressure (1 bar)	Dead‐end cell	1000 ppm Divalent ions	5.01	5.01	> 90%	–	^[^ ^84d^ ^]^
rGO‐TA^e)^	150 nm	External Pressure (1 bar)	Vacuum Filtration	1000 ppm Organic Dyes	2972	2972	–	100% MB	^[^ ^74^ ^]^
rGO‐TA	150 nm	External Pressure (1 bar)	Vacuum Filtration	1000 ppm Organic Dyes	2547	2547	–	76% Methyl Blue	^[^ ^74^ ^]^
Ultrathin rGO Membrane	22 nm	External Pressure (5 bar)	Dead‐end cell	0.02 m MB solution	109	21.81		99.2% MB	^[^ ^38a^ ^]^
Ultrathin rGO Membrane	53 nm	External Pressure (5 bar)	Dead‐end cell	0.02 m NaCl	–	–	≈40%	–	^[^ ^38a^ ^]^
HI‐reduced ultrathin rGO Membrane	100 nm	Osmotic Pressure	FO Cross‐flow test	2 m NaCl	57	–	0.2 mol m^−2^ h^−1^	–	^[^ ^88^ ^]^
GO/rGO	5 µm	Osmotic Pressure (3 m sucrose)	FO U‐shape cell	0.1 m NaCl	0.5	–	97%	–	^[^ ^52^ ^]^
GO/rGO	5 µm	Osmotic Pressure (3 m sucrose)	FO U‐shape cell	0.1 m NaCl	2.5	–	94%	–	^[^ ^52^ ^]^
GO/0FLG	–	External Pressure (50 bar)	RO Cross‐flow test	0.034 m NaCl	336	6.7	26%	–	^[^ ^89^ ^]^
GO/60FLG	–	External Pressure (50 bar)	RO Cross‐flow test	0.034 m NaCl	23.2	0.46	85%	–	^[^ ^89^ ^]^
Single‐layer GNM/SWNT hybrid membrane	50 nm	Osmotic Pressure (2 m sucrose)	FO Cross‐flow test	0.5 m NaCl	–	20	98%	–	^[^ ^92^ ^]^
Single‐layer GNM/SWNT hybrid membrane	50 nm	External Pressure (5 bar)	RO Cross‐flow test	2000 ppm NaCl	≈500	≈100	87%	–	^[^ ^92^ ^]^
TiO_2_@rGO nanocomposite Membrane	≈400 nm	External Pressure (8 bar)	RO Cross‐flow test	0.5 g L^−1^ Rose Bengal	≈28	≈3.5	–	> 97% Rose Bengal	^[^ ^95a^ ^]^
GO‐incorporated Polyamide Membrane	≈100 nm	External Pressure (15 bar)	RO Cross‐flow test	2000 ppm NaCl	≈25	≈1.7	≈88%	–	^[^ ^95c^ ^]^
GO/MIL‐88A(Fe)	≈10 µm	External Pressure (1 bar)	Vacuum Filtration	10 mg L^−1^ MB solution	28.7	28.7	–	99.6% MB	^[^ ^96a^ ^]^
GO/UiO66	≈200 nm	Osmotic Pressure	FO Cross‐flow test	2 m NaCl	29.16	–	0.22 mol m^−2^ h^−1^	–	^[^ ^97^ ^]^
GO/ZIF‐8	105 nm	External Pressure (1–7 bar)	RO Cross‐flow test	0.1 g L^−1^ MB solution	60	60	–	≈100% MB	^[^ ^73^ ^]^
GO/Porphyrin	N/A	External Pressure (8 bar)	FO Cross‐flow test	0.034 m NaCl	≈7	≈0.88	25%	N/A	^[^ ^101a^ ^]^
GO/Porphyrin	N/A	External Pressure (8 bar)	FO Cross‐flow test	0.014 m Na_2_SO_4_	≈8.5	≈1.06	89%	N/A	^[^ ^101a^ ^]^
Voltage‐gated MXene Membrane	1.5 µm	Electric Potential (−0.5 V)	Dead‐end Filtration	10 ppm MB	–	5.6	–	99.6% MB	^[^ ^61a^ ^]^
Voltage‐gated MXene Membrane	1.48 µm	Electric Potential (−0.6 V)	FO U‐shape cell	0.6 m NaCl	–	–	3 × 10^−3^ mol m^−2^ h^−1^	–	^[^ ^61a^ ^]^
Voltage‐gated MXene Membrane	1.48 µm	Electric Potential (0.4 V)	FO U‐shape cell	0.6 m NaCl	–	–	0.26 mol m^−2^ h^−1^	–	^[^ ^61a^ ^]^
Surface‐charged GO Membranes	≈100 nm	External Pressure (2 bar)	Dead‐end cell	500 ppm MgCl_2_	–	51.2	93.2% MgCl_2_	–	^[^ ^102^ ^]^
Surface‐charged GO Membranes	≈100 nm	External Pressure (2 bar)	Dead‐end cell	500 ppm Na_2_SO_4_	–	56.8	93.9% Na_2_SO_4_	–	^[^ ^102^ ^]^

^a)^ Ion rejection rates are in the unit of %, while reverse ion fluxes are in the unit of mol m^−2^ h^−1^

^b)^ MB: Methylene blue

^c)^ SA: Alginate polysaccharide

^d)^ HPEI: Hyperbranched polyethylenimine

^e)^ TA: Tannic acid.

The requirements of the membrane structural design become more complicated when ion transport is involved. It was found that ion permeation rates can be several thousands of times faster than those predicted from traditional ion diffusion model, demonstrating similar transport behavior as that of water through hydrophobic graphene nanocapillaries (<2 nm), which can also be attributed to a capillary‐like pressure between ions and the capillary walls. This phenomenon should be beneficial for the improvement of ion selectivity. However, the phenomena of ion dehydration and ionic Coulomb blockade have also been observed. On one hand, the size of dehydrated ions can be as small as ≈1 Å at least along one dimension, which makes the rejection of those dehydrated ions unrealistic in practice by size exclusion mechanism. Hence, the membrane structural design should consider the prohibition of ion dehydration. On the other hand, ionic Coulomb blockade may cause ion fouling on the NF membranes. The fine modulation of pore chemistry may be required in preventing this undesired blockage of membrane pores. These considerations should also be applied onto single‐layered 2D material NF membranes and MOF‐2D material composite membranes.

The effect of electric fields on water and ion transport through nanocapillaries have been preliminarily explored. Ion dehydration has been repeatedly observed in this case, and the abnormal phenomenon, i.e., the enhancement of ion transport by electrostatic manipulation under the nanoconfinement (<2 nm), has been found and ascribed to the effect of ion–ion correlations. Direct electrostatic manipulation of ions across nanocapillaries provides us with a diversified toolbox for the improvement of the overall performance of 2D material NF membranes. In the case of ion fouling on the NF membranes induced by ionic Coulomb blockade, adding external potential may be a promising method to solve the problem. The improvement of antiswelling property can also be achieved through the electrostatic interactions, e.g., the cationic control of interlayer spacing in GO and MXene membranes.^[^ ^78^, ^81^ ^]^ The use of electrostatic repulsion to exclude ions has also been successfully applied onto surface‐charged GO membranes.^[^ ^102^ ^]^ However, the strength of using electric field/surface charge in improving the overall performance has not been fully explored. Membrane structural designs that can take full advantage of the abnormal phenomena observed for the electrostatic manipulation of ion transport through sub‐2‐nm capillaries, e.g., ion–ion correlations, are highly demanded, which requires considerable efforts in the future.

Based on the understandings and discussion shown above, we prospect that the optimization of membrane structures may be realized through the right design of several sublayers in the membrane with different functionalities. Note that this is fundamentally different from multilayer architectures shown in Section 3.1.3. As an example, the design of a thin hydration sublayer on the surface of the restacked 2D material NF membranes mainly with 2D hydrophobic graphene nanocapillaries might be an effective way to prevent ion dehydration. Other promising designs for sublayers are those which can integrate size exclusion mechanism with like‐charge exclusion mechanism, or which can integrate size exclusion mechanism with the advantages of adding external electric potential, in the same membrane. The types of the sublayers may be adjusted according to different requirements in real industrial applications. More importantly, the right combination of the sublayers may promote new synergistic effects in improving overall performance, as illustrated in surface‐charged GO membranes.^[^ ^102^ ^]^

Last but not least, theoretical descriptions of water and ion transport within highly confined nanocapillaries (<2 nm) needs further development.^[^ ^107^ ^]^ This could be achieved through the detailed analyses of microscopic forces between water/ion and capillary walls/pores under different external conditions (e.g., externally applied electric potential or hydrostatic pressure). However, colloidal forces are crowded in the case of ions under sub‐2‐nm capillaries, ions have various sizes and valence, capillary walls/pores have different functionalities and surface charge, and more challenging is that ions can undergo ion dehydration or ionic Coulomb blockade when passing by. All these factors add complexities to the descriptions of water and ion transport through the highly confined nanocapillaries. Reasonable assumptions should be made according to different situations to simply the theoretical expressions, which should then be followed by experimental validated.

Note that, although the rejection of organic dyes is not as challenging as that of ions in water purification, all the strategies discussed above may be applied to the rejection of organic dyes if necessary. It is further projected that, combining experimental and simulation work with theoretical studies, more unusual and useful phenomena are expected to be predicted, observed, and explicated in the near future, guiding the rational membrane structural designs and the continuous development of 2D material NF membranes not only for water purification but also for energy storage, salinity gradient energy harvesting, field‐effect transistors with gate dielectrics of ionic liquids, etc.

## Conflict of Interest

The authors declare no conflict of interest.
